# Conceptualizing Young People's Experiences of Climate Change Awareness: A Narrative Review

**DOI:** 10.1111/nyas.70114

**Published:** 2025-10-26

**Authors:** Daniella Watson, Teaghan Hogg, Tara Crandon, Ans Vercammen, Chloe Watfern, John Jamir Benzon Aruta, Fiona Charlson, Emma Louise Lawrance

**Affiliations:** ^1^ Institute for Global Health Innovation Imperial College London London UK; ^2^ Grantham Institute for Climate Change and the Environment Imperial College London London UK; ^3^ Discipline of Psychology, Faculty of Health University of Canberra Canberra Australia; ^4^ Queensland Centre for Mental Health Research Wacol Australia; ^5^ Institute for Social Science Research University of Queensland Brisbane Queensland Australia; ^6^ School of Population Health Curtin University Perth Australia; ^7^ Black Dog Institute Randwick New South Wales Australia; ^8^ Department of Psychology De La Salle University Manila Philippines

**Keywords:** climate anxiety, climate change, climate psychology, eco‐anxiety, mental health, young people

## Abstract

There are a myriad of ways that young people experience and respond to awareness of the climate crisis. The way these experiences and their potential impacts on mental health are conceptualized is not yet clear or consistent for this emerging field of research. Consolidating emerging concepts, definitions, and theories can help to focus future research, policy, and interventions. We conducted a narrative review of academic literature covering climate change and mental health research focusing on young people. We searched Web of Science for English‐language reviews published until June 27, 2024, and primary data published between January 2023 and June 2024. We extracted relevant concepts and created categories of concepts. Artificial intelligence (AI) technologies (ASReview and ChatGPT) assisted papers screening and categorization, with human validation from global experts. We identified 93 relevant articles describing 173 terms for young people's experiences of climate change awareness; 52 coping strategies; and 62 frameworks, models, and theories with varying and overlapping definitions. We present and categorize the most frequent. This narrative review provides a foundational guide to the complex field of climate psychology, focusing on young people. We outline a seven‐phase roadmap to further explore and consolidate understandings of emerging concepts and to map their interrelationships.

## Graphical abstract

Young people experience climate change awareness in diverse ways, yet conceptual clarity in this emerging field remains limited. We conducted a narrative review of 93 relevant articles, identifying 173 concepts, 52 coping strategies, and 62 organizing frameworks. Using AI‐assisted screening and expert validation, we categorized key concepts. We propose a seven‐phase roadmap to guide future research, emphasizing the need for consolidated definitions, theoretical grounding, and culturally inclusive methodologies in climate psychology.

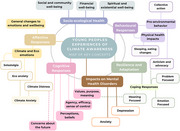

## 1 Introduction

Climate change has profound adverse effects on young people's mental health. The climate crisis not only exacerbates acute and chronic existing mental health conditions but also contributes to the development of new mental health problems among young people [1]. Young people's mental health can be affected by their direct and/or indirect experiences of climate change, including increased exposure to climate hazards; increased awareness of climate change–related threats without sufficient action from leaders; disruptions to social and cultural practices, socioeconomic, and political conditions; and the exacerbation of structural and environmental inequalities and injustices [2, 3, 4, 5]. Without concerted action on climate change, rates of mental ill‐health will increase in young people, compromising their overall health and well‐being [5]. Action on climate change is urgently needed to safeguard the mental health of young people. It is also necessary to develop evidence‐based climate‐informed interventions that support young people's mental health as the climate worsens. A first step to developing these interventions is to understand how young people respond to and cope with the climate crisis, including awareness of current and future threats.

Climate psychology, an emerging interdisciplinary research area, is making headway in describing and measuring the wide‐ranging psychological responses to the climate crisis and their relationships with behavior and mental health impacts [6]. The Climate Psychology Alliance (https://www.climatepsychologyalliance.org/—home page) defines climate psychology as “concerned with the emotions, and the social and mental processes that have contributed to the ecological and climate crisis, and our responses and processes of adaptation to it.” This field emphasizes the significance of emotions and psychological responses to awareness of the climate crisis as they appear in individual lives and group dynamics [7]. The climate psychology field also examines how identities and social discourse help people to make sense of the climate crisis and facilitate or hinder their engagement in climate action [7].

Research on climate change and mental health has proliferated in recent years. Romanello et al. [8] note that publications in the field have seen a 210% increase since 2016. This dramatic increase has been accompanied by a vast array of emerging terminology, theoretical frameworks, and metrics attempting to describe and explain the many ways in which people are responding psychologically to the climate crisis [3, 9, 10, 11, 12], leading to potential confusion and overwhelm for the field. Niedzwiedz et al. [13] provide a glossary of the key concepts and mechanisms through which climate change can influence mental health, examining responses across multiple levels—from individual emotional reactions to broader political actions. The glossary is a fantastic start at mapping out the broad range of ways that people respond to climate change in general; however, the *affective responses* listed in the glossary were limited. Elsewhere in the literature, commonly used constructs such as *eco‐anxiety*, *climate anxiety*, and *ecological grief* (among others) are currently poorly and heterogeneously defined and measured, sometimes used interchangeably, and with their interrelations uncertain.

A case in point here is the interchangeable use of eco‐anxiety and climate anxiety [14], an example of the so‐called Jingle‐jangle fallacy, coined by Kelley [15] to describe situations where two scales with similar names measure different constructs (jingle fallacy), or where two scales with seemingly dissimilar labels measure similar constructs (jangle fallacy). For example, there are a variety of metrics that measure *climate anxiety* [16], *climate distress* [17, 18], *climate worry* [19], and *eco‐anxiety* [20]. These scales have overlaps, and there remains ambiguity from the titles of what aspects of the construct they are measuring, including behavioral, affective, cognitive, and physical factors and their functional impact, and the severity of the responses they measure. A study of 2834 youth from the United States produced different results for the Hogg Eco‐anxiety Scale [19] and Reser's Climate Distress Scale [20] across the sample, with distress being generally moderate to high, whereas eco‐anxiety was low [21]. Examination of the eco‐anxiety scale indicates that it provides a measure of a more severe level of impairment, or incursion of psychological responses to the climate crisis into everyday life and functioning. The measure of climate distress, on the other hand, seems to assess a less severe and less disruptive sense of general unease or concern, which is not apparent from the name of the scales alone, leading to potential misinterpretation when comparing results across studies.

Likely contributing factors are the natural evolution of research methods, the evolving and emerging nature of these complex experiences that the metrics seek to capture, and researchers from various fields using diverse practices grounded in distinct theoretical underpinnings. Although a plurality of perspectives is vital, the rapid increase in competing definitions and metrics is leading to conceptual confusion and inconsistency in terminology across a nascent transdisciplinary field [22].

Although uncertainty and fluidity are characteristic of an emerging field, it is important that the expansion of research and the pace of the field's growth do not come at the expense of consolidating our collective understanding. Researchers must be able to distinguish between true experiential variations in psychological responses to the climate crisis and inconsistencies due to conceptual or methodological variability. Without consistent, consolidated definitions of psychological responses to the climate crisis, their links to health and behavior, and their relationships with each other, it will be difficult to consistently measure and compare evolving attributes of climate change awareness and evaluate effective support tools and coping strategies [4].

Developing a reliable, valid, and operational measure of a psychological phenomenon is challenging without a consistent and comprehensive theoretical foundation [23]. Moreover, although climate psychology adopts theories, models, and frameworks from related disciplines, current climate psychology scales and interventions have been developed with limited theoretical bases. For example, climate anxiety has been researched for several years, with the application of influential and frequently used scales such as the Climate Change Anxiety Scale [16] published in 2020. It was not until 2024 that Crandon et al. [14] provided a theoretical perspective on how climate anxiety is influenced by and interacts with different layers of a young person's socioeconomic environment. Recently, van Valkengoed and Steg [24] developed the *climate anxiety compass* to map the solution space for climate anxiety, which classifies support or coping strategies within three dimensions: (1) problem‐focused or emotion‐focused, (2) mitigation or adaptation to the mental health impacts of the climate crisis, and (3) individually oriented or collectively oriented. As a developing field, climate psychology needs to draw on relevant expertise and related disciplines—to gather the required data to validate and refine core concepts and their relationships in a coherent theoretical framework. The climate psychology field is now at a critical point, however, where the burgeoning research requires consolidation and clarity to ensure its value in aiding our collective understanding of the dynamic and evolving psychological responses to the unfolding climate crisis.

We recognize that there are a number of phases required to fully consolidate, validate, and refine core concepts and their relationships in a coherent theoretical framework. In the discussion section of this article, we propose a roadmap of seven phases needed for the field to rigorously and appropriately consolidate definitions of key concepts and map relationships between concepts. The seven phases include (1) mapping the concepts in the literature, (2) engaging in interdisciplinary dialogue, (3) mapping interrelationships between key concepts, (4) adopting machine learning to build the framework, (5) investigating risk and protective factors, (6) testing and validating concepts, and (7) disseminating. To fulfill the first phase of this seven‐phased roadmap, we conducted a narrative review of the climate and mental health literature with a specific focus on the experiences of young people in response to climate change awareness. The aim of this narrative review is to take the initial steps towards greater conceptual clarity through a structured approach, by providing an overview of the variety of key concepts and different definitions and frameworks that have shaped research on young people's experiences of the climate crisis, including highlighting the patterns of current use across the literature. The focus on young people is because (1) the climate change and mental health literature to date has largely focused on young people as (2) they have particular vulnerabilities to the mental health impacts of climate change and unique sources of resilience. These factors arise from their developmental stage [25], the larger role that the climate crisis will play over their lifetime [26], their heightened awareness of environmental issues [27], and the significant role they will play in current and future climate action, adaptation, and mitigation [28].

## 2 Materials and Methods

A narrative review enables a broad overview of the climate change and mental health literature. Compared to systematic and scoping reviews, a narrative review allows for researchers’ subjective examination and critique of an entire body of literature, where a topic's current status, evidence, and advancements can be explored [29].

Our literature search focused on young people and their experiences of and responses to climate change awareness and the impacts on their mental health and well‐being (Table 1). This review is novel and differs from other reviews such as Crandon et al. [30], Ma et al. [31], and Olsen et al. [32] as the review systematically focuses on the broad spectrum of young people's experiences and responses to climate change awareness, not just eco‐anxiety or climate anxiety or clinical diagnoses.

**TABLE 1:** Search terms used in Web of Science to identify relevant papers.

Key words	Search terms
Climate change	Climate change OR global warming OR climate crisis OR climate emergency OR climate disaster
Mental health	Mental health OR well‐being or wellbeing OR emotional health OR psychological health OR solastalgia OR psychoterratic syndrome* OR eco*emotions OR ecological grief OR eco*anxiety OR climate*anxiety OR climate emotion* OR climate grief OR climate anger OR climate rage
Young people	Young people OR youth OR adolescents OR young adult OR adolescent* OR teen* OR student*

**FIGURE 1:**
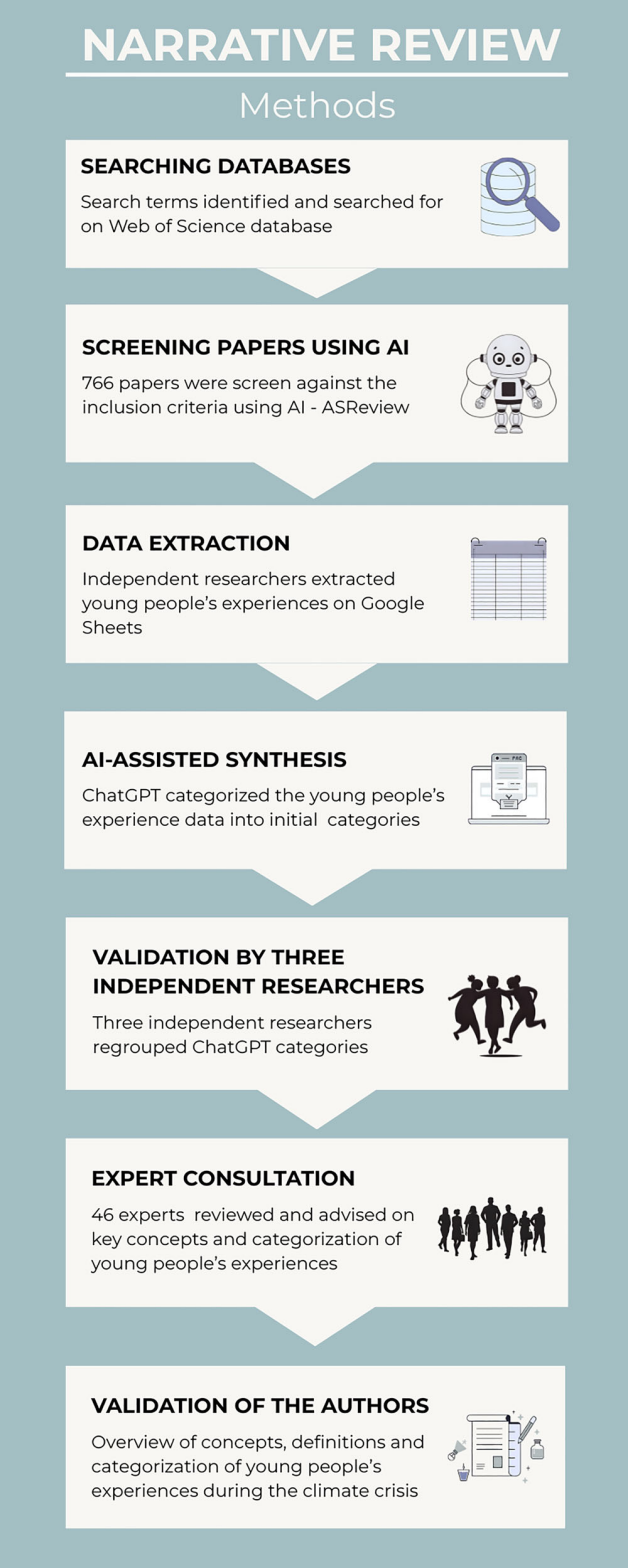
Flowchart of methods used in the narrative review. AI, artificial intelligence.

**FIGURE 2:**
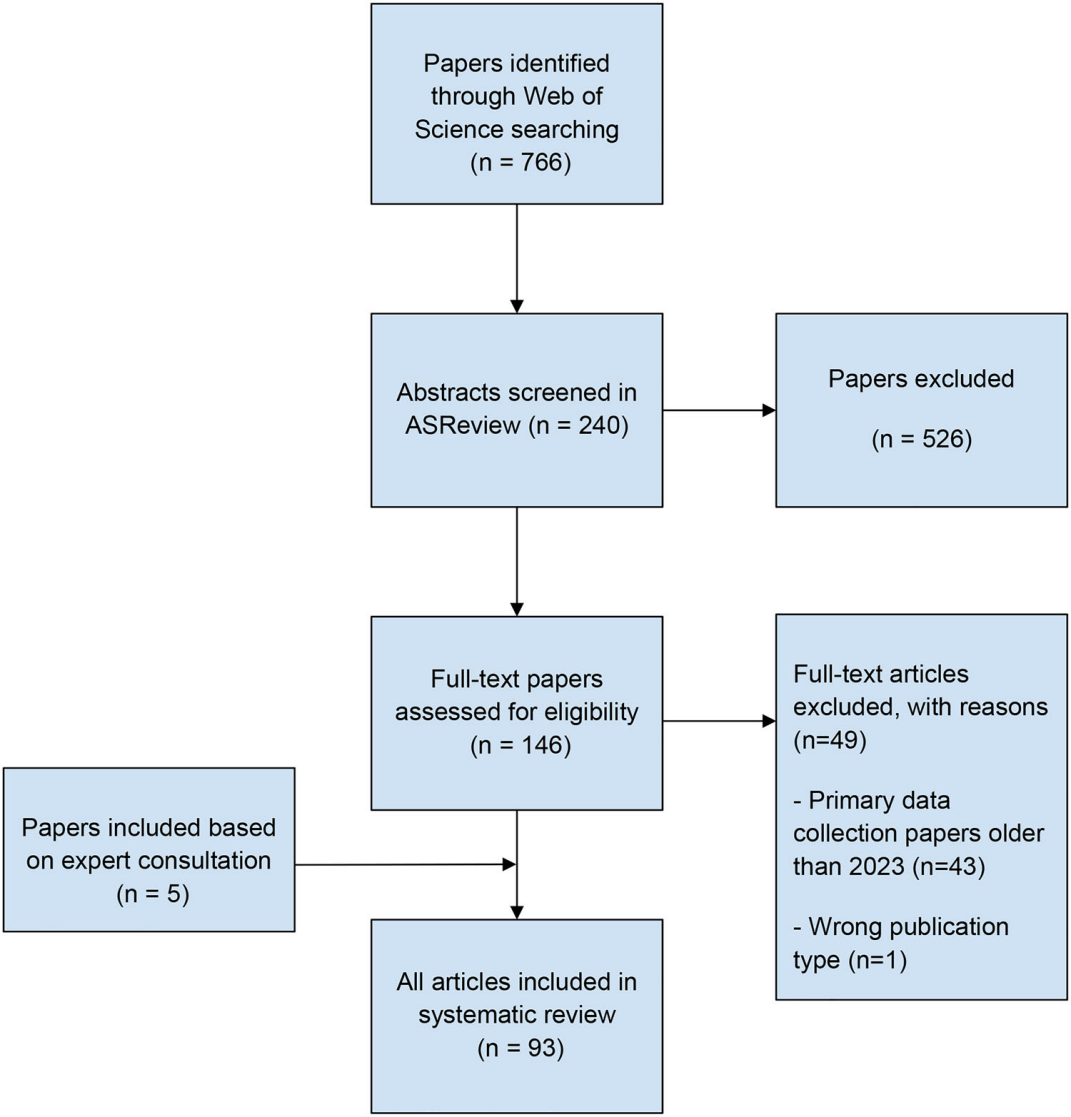
Flowchart of paper selection.

A search was performed on the Web of Science database, searching all relevant English‐language articles published prior to June 27, 2024 (inclusion and exclusion criteria are available in Table 2). Search terms included those to capture concepts associated with climate change, and those associated with mental health and well‐being, with a specific focus on concepts related to psychological responses to climate change awareness. Studies only focusing on exposure to climate events without participants attributing the event to being influenced by the climate crisis were excluded. Search terms were generated on the basis of previous reviews [3, 33] and recommendations from a librarian and the research team (Table 1). A total of 766 papers were retrieved. The papers were automatically organized by Web of Science into the highly cited papers (most citations; *n* = 5), review articles (*n* = 27), and all other papers. We made the decision, with support from our librarian, to include the highly cited papers and reviews and then screen all other original research articles published between 2023 and 2024 to assess their suitability for inclusion. This decision was taken on the basis that the identified review papers would capture most of the relevant papers before 2023, and we wanted to focus on the most recent evidence and debates in a rapidly evolving field. Figure 1 shows the method process used in this review, whereas Figure 2 shows the flowchart of paper selection (Section 3).

**TABLE 2:** Inclusion and exclusion criteria

Adapted from PICO [133]	Inclusion criteria
Publication type	Original peer‐reviewed research, review papers, commentaries, books, academic reports
Population	Young people—people aged 10–25 years [29], or studies with mean ages within that range Adults who care or work with young people
Climate change terms	Climate change terms (see Table 1) Only include papers assessing “natural disasters” if they are considered in the context of climate change awareness
Outcomes	Climate awareness, psychological (including emotional) responses to climate change, mental health outcomes of climate awareness
Additional limits	English‐language articles No time limit

### 2.1 Inclusion and Exclusion Criteria

Papers published between 2023 and 2024 were screened on ASReview; “an open‐source tool that employs active learning and multiple machine learning models to assist researchers in making inclusion and exclusion judgments” [34, p. 2]. We trained ASReview on the inclusion and exclusion criteria (Table 2). The software then reshuffled the unreviewed articles so that the most relevant were ranked higher in the dataset [34, 35]. At the point where 20% of the papers had been screened (*n* = 153), no additional relevant studies were identified. ASReview indicated that all relevant papers had likely already been included. We applied a stopping rule and concluded screening after 31% of the papers (*n* = 240). This decision aligns with the stopping criteria proposed by Boetje and van de Schoot [35] and van de Schoot et al. [34], whose simulation studies suggest that all eligible papers can typically be identified after screening 8%–33% of the dataset, provided that at least 50 consecutive papers yield no new inclusions. Our stopping point—after screening 31%—is consistent with these recommendations.

### 2.2 Data Extraction

Google Sheets was used for data extraction to allow multiple researchers to work simultaneously. Three researchers (D.W., T.H., T.C.) had multiple meetings to ensure consistency of data extraction. Data extracted included the paper's key details (authors, paper title, publication year, and journal), context of the paper (aims, population, geographical focus, climate change–related exposures), methodology (methods, sample size, analysis methods), mental health and psychological outcomes (see below), main results, key discussion points, strengths, and limitations.

To appropriately extract the large number of relevant concepts, we initially extracted the psychological impacts and responses to climate change awareness highlighted in the articles, such as climate anxiety. This process highlighted the large range of psychological responses and related concepts documented in the literature on, more generally, young people's experiences of climate change awareness, and hence, we subsequently expanded the data extraction to further include the following categories: (1) frameworks/models/theories (frameworks) organizing the concepts, (2) psychological and emotional responses, (3) behavioral responses, (4) cognitive responses, (5) climate change and mental health metrics, (6) coping strategies, (7) risk factors, and (8) protective factors. This approach was taken to structure the enormous number of emerging concepts and frameworks in the literature. During data extraction, we sorted concepts under these eight initial categories. Extraction included copying the exact term used in each paper for sufficiently frequent concepts such as “climate anxiety.” For idiosyncratic descriptions of relevant young people's experiences, such as different ways to describe impacts on future‐oriented concerns or decision‐making, for example, concerns about future safety and resources due to the climate crisis or concerns about having children in the climate crisis, we used convergent concepts—for example, “future outlook”—for manageability of the volume of concepts and descriptions. For each term, we extracted the definition or description used for these concepts and the reference to the original source. By referring to the original sources, we included climate psychology papers from beyond 2023–2024 and in all populations (beyond those focused on young people). The data extraction categories were later synthesized and evolved into alternative ways of categorizing and interpreting the data.

### 2.3 Data Synthesis Phases

#### 2.3.1 Phase 1: Artificial Intelligence (AI)‐Assisted Synthesis

To make sense of hundreds of extracted data points, we applied AI and natural language processing (NLP) technology ChatGPT. Biswas [36] advocated for using ChatGPT for data analysis and interpretation for large datasets related to climate change, and Michie et al. [37] used AI and machine learning to develop an ontology of behavior change interventions. For example, we asked ChatGPT “How would you categorize these frameworks, models and theories related to climate change and mental health?” based on the list of concepts identified from the data extraction in that category. We asked ChatGPT the same question for each of the following: (1) frameworks/models/theories, (2) psychological and emotional responses, (3) behavioral responses, (4) cognitive responses, (5) climate change and mental health metrics, (6) coping strategies, (7) risk factors, and (8) protective factors. ChatGPT created separate subcategories for each of the eight categories of extracted data. The AI subcategories provided the first iteration of the subcategories presented in this narrative review.

#### 2.3.2 Phase 2: Validation by Three Independent Researchers

As advised by an expert in the application of AI for the creation of mental health ontologies [38], we employed numerous rounds of human validation. The three researchers involved in data extraction separately restructured the ChatGPT subcategories and met to reach consensus. For example, we collapsed the categories that we extracted data from the outcomes on (categorization not based on AI), the (1) psychological and emotional responses, (2) behavioral responses, and (3) cognitive responses into a single concept/term, into *young people's experiences*, signifying their broad range of responses to awareness of the climate crisis. We believed that the term *responses* did not fully encompass young people's experiences with the climate crisis. Additionally, experiences such as effects on physical health and relationships that were highlighted in the literature appeared somewhat outside of the scope of psychological, emotional, behavioral, and cognitive responses. We found that *young people's experiences* better captured the wide range of impacts and reactions that young people encounter during awareness of the climate crisis.

#### 2.3.3 Phase 3: Expert Consultation

To further validate our findings and interpretations from the data extraction, we organized two expert meetings with 46 experts in the field of climate change and young people's mental health. Experts spanned research (*n* = 28), civil society (*n* = 10), and clinical practice (*n* = 7) across 16 countries (Australia (*n* = 11), Brazil (*n* = 3), Canada (*n* = 2), Finland (*n* = 1), Germany (*n* = 1), Ireland (*n* = 1), Italy (*n* = 2), India (*n* = 1), the Netherlands (*n* = 1), New Zealand (*n* = 1), the Philippines (*n* = 1), Sweden (*n* = 2), the United Kingdom (*n* = 15), the United States (*n* = 3), and unknown sector and country (*n* = 1)). Experts reviewed and revised the results and advised on the best way to present them to benefit the field of climate psychology. The detailed expert consultation will be reported elsewhere, especially regarding highlighted gaps in approaches to climate psychology research, and we report here that the experts recommended to generate knowledge from the global south and with indigenous communities and the need to work transdisciplinary. Additionally, the experts specifically recommended distinguishing subcategories related to mental health and well‐being, mental disorders, general emotions, and climate emotions, which is also incorporated into our synthesis.

#### 2.3.4 Phase 4: Validation of the Authors

The data and expert validation were reviewed and updated during a meeting with the coauthors of this review (A.V., C.W., D.W., J.J.B.A., T.C., T.H.) and in separate meetings with F.C., T.C., T.H., E.L., and A.V. Due to the large volume of data extracted, a decision was made to focus on the following categories: young people's experiences, coping strategies, and frameworks.

## 3 Results

### 3.1 Description of the Identified Papers

A total of 93 papers [6, 9, 10, 16, 30, 31, 32, 39, 40, 41, 42, 43, 44, 45, 46, 47, 48, 49, 50, 51, 52, 53, 54, 55, 56, 57, 58, 59, 60, 61, 62, 63, 64, 65, 66, 67, 68, 69, 70, 71, 72, 73, 74, 75, 76, 77, 78, 79, 80, 81, 82, 83, 84, 85, 86, 87, 88, 89, 90, 91, 92, 93, 94, 95, 96, 97, 98, 99, 100, 101, 102, 103, 104, 105, 106, 107, 108, 109, 110, 111, 112, 113, 114, 115, 116, 117, 118, 119, 120, 121, 122, 123] were included in the narrative review (Figure 2, Tables S1 and S2). The final selection included 30 review papers, 36 papers using quantitative methods, 14 papers using qualitative and participatory methods, 5 opinion pieces, 6 intervention studies, and 2 papers using mixed methods.

Two‐thirds of the studies that specified the countries of participants were conducted in high‐income countries (*n* = 41, 66%, Table S1), with a third of studies focusing on low‐middle‐income countries (*n* = 21, 34%, Table S1). Seven studies included countries spanning diverse income contexts, and 21 studies did not specify a particular country or context of interest. Six studies included or considered Indigenous and First Nations people, a group who are underrepresented in the literature. Studies that worked with groups of young people ranged from children aged 0–10 years (*n* = 2, 2%), adolescents aged 10–19 years (*n* = 17, 18%), young adults aged 19–24 years (*n* = 17, 18%), young people across age ranges (*n* = 22, 25%), studies referring to university students without an age range (*n* = 8, 9%), professionals working with young people (*n* = 4, 5%), young person and professionals together (*n* = 3, 3%), young person and their parents (*n* = 1, 1%), and studies that did not define “young person” (*n* = 19, 19%).

### 3.2 Climate Change–Related Events

Most papers studied climate change–related events in general or concerned themselves with a range of discrete impacts rather than one single event. Only five studies specified exposures perceived to be climate change–related, namely, air pollution [39], bushfires [40, 41], degradation of marine environments [42], and floods [43]. Studies with more general climate change–related events referenced a range of different combinations of acute exposures, that is, climate change–related hazards, including floods, hurricanes, thunderstorms, cyclones, tornadoes, earthquakes, typhoons, tsunamis, volcanoes, and landslides. Extreme weather conditions encompassed heat, heatwaves, high‐temperature weather, snowstorms, humidity, and excessive rain. The inclusion of fires as a climate impact encompassed forest fires, bushfires, and wildfires. Studies also referenced more slow‐onset climate change effects such as sea‐level rise, increased water levels, melting sea ice, loss of shoreline, drought, and desertification. Environmental issues that were associated with climate change involved land pollution, air quality, ocean pollution, deforestation, loss of biodiversity, influx of invasive species, and mass extinction of animals.

### 3.3 Climate Psychology Concepts and Frameworks

We identified 173 different concepts used to describe young people's experiences of climate change awareness, 52 coping strategies, and 62 frameworks.

#### 3.3.1 Young People's Experiences

**FIGURE 3:**
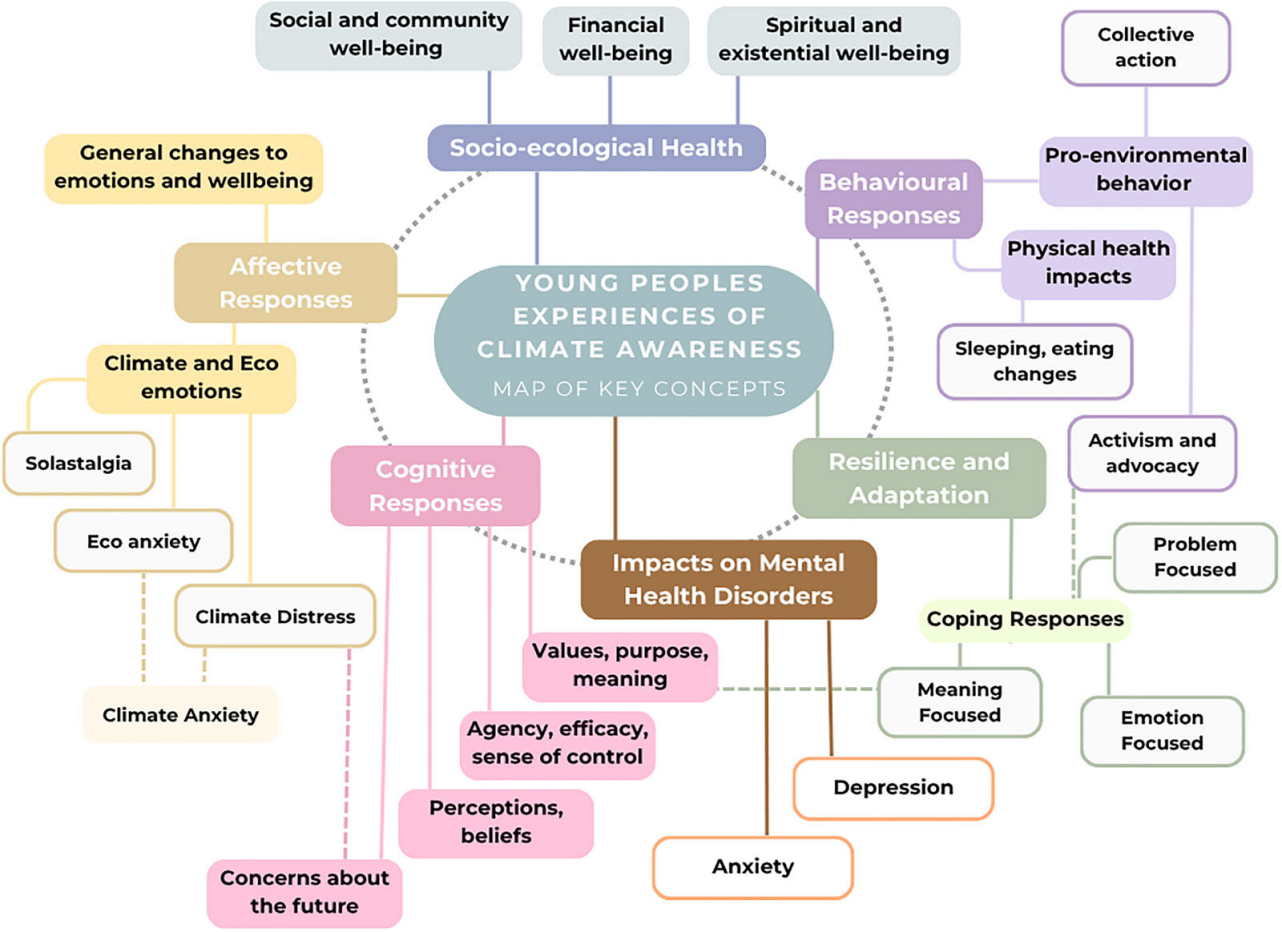
**Hypothesis map of the key concepts and developed categories and subcategories of young people's experiences of climate change awareness**. The figure illustrates the narrative review authors’ interpretation of the vast array of concepts related to young people's experiences of climate change awareness and how these concepts can be categorized into six interrelated categories and subcategories. The solid lines represent umbrella terms and concepts that fall under that umbrella term. For example, the subcategory “climate and eco‐emotions” falls under the category of “affective responses,” whereas the subcategory for “coping responses” falls under the category of “resilience and adaptation.” The dotted lines indicate where something reflects one illustrative type of a given concept. For example, thinking about “values, purpose, meaning” is one type of “meaning‐focused” coping response, and “climate anxiety” is one type of “eco‐anxiety.”

“Young people's experiences” in this review were inclusive of the full spectrum of physical, emotional, cognitive, functional, and behavioral responses to, and impacts arising from, climate change awareness that were identified in the literature. To map the 173 experiences, we grouped them into six subcategories (Figure 3, Table 3). Due to the relative lack of evidence in the literature about the relationship between concepts, made more complex by the fuzziness of the concept bounds, the authors of this narrative review attempted to consolidate emerging insights by categorizing the concepts and illustrating potential interrelationships between concepts based on their own interpretation of the literature. We advise how the field can empirically develop robust consolidation of definitions, categories, and the interrelationships between both concepts and categories in a seven‐phase roadmap in the discussion section. The most frequent young person's experience documented was “eco‐anxiety,” a term found in 41 papers. Many of the more frequently documented experiences, such as eco‐anxiety and climate anxiety, had highly variable definitions (Table 4). Even the established term “depression” had no clear definition in any of the nine papers that reported on it. Some papers reported a list of emotions that arise in the context of climate change awareness based on speculation within discussion sections rather than from empirical evidence within the results sections.

**TABLE 3:** Categories and subcategories used to organize “young people's experiences” of climate change awareness, their subcategory descriptions, and illustrative example concepts that belong to the subcategories from the papers in our sample.

The six categories to organize dimensions of the identified “young people's experiences” of climate change awareness	Subcategories	Subcategory descriptions	Example concepts
Affective responses	Climate and eco emotions	Both discrete and multifaceted psychological responses and reactions to the awareness of ecological problems, including the climate crisis	Eco‐anxiety Climate hope Climate dread
	General changes to emotions and well‐being	General emotional reactions and factors relevant to people's quality of life	Worry Fearful Hope Optimism
Cognitive responses	Values, purpose, meaning	Personal values, moral frameworks, sense of identity, and life purpose includes internal conflicts (e.g., cognitive dissonance), ethical distress (e.g., moral injury), and the search for meaning, all of which shape how they make sense of and respond to ecological threats	Cognitive dissonance Moral injury Identity
	Agency, efficacy, sense of control	Young people's perceived ability to influence or respond to the climate crisis, either individually or collectively. This includes feelings of autonomy, empowerment, and individual, collective and environmental efficacy, which shape their motivation, engagement, and resilience in the face of ecological challenges	Autonomy Empowerment Collective‐efficacy
	Perceptions, beliefs	Young people interpret the reality, causes, and consequences of the climate crisis. This includes varying levels of climate risk perception, uncertainty, and in some cases, denial or disengagement. These perceptions influence emotional responses, motivation, and engagement with climate action	Climate denial Risk perceptions Feelings of uncertainty
	Concerns about the future	Thoughts and anticipations about the long‐term impacts of the climate crisis on personal, societal, and planetary futures. This includes worries about environmental degradation, uncertainty about life plans, and challenges in envisioning or preparing for the future. These concerns can influence young people's mental health, decision‐making, and sense of stability	Concerns about the future Future planning Anticipating a significant loss of biodiversity
Impacts on mental disorders	N/A	A mental disorder is characterized by a clinically significant disturbance in an individual's cognition, emotional regulation, or behavior. It is usually associated with distress or impairment in important areas of functioning [125]	Post‐traumatic stress disorder Depression Anxiety
Behavioral responses	Pro‐environmental behavior	The actions and practices individuals or groups adopt to reduce their environmental impact and support sustainability. Such behaviors may also serve as coping strategies, as further explored under the category of “Activism and Advocacy”	Climate action Activism Collective action Support for climate policies
	Physical health impacts	Encompass concepts related to changes in individuals’ physical health, including alterations in sleep patterns, eating habits, energy levels, and the onset of physical ailments or illnesses. These responses often overlap with shifts in mental health and overall well‐being	Changes to sleeping habits Changes to eating habits Increased hospital admissions
Socio–ecological health	Social and community well‐being	The interconnected psychological and social responses individuals and communities experience in relation to their broader environment. It encompasses aspects of social health, such as the quality of personal relationships and the well‐being of communities	Impact on community well‐being Relationships Concern for loved ones
	Financial well‐being	Concepts related to the psychological responses to changes in financial well‐being of people, families, and communities both at the personal and collective levels	Financial strain Perceived financial (in)security Financial decisions
	Spiritual and existential well‐being	Concepts such as people's values, spirituality, sense of purpose and meaning are also central, reflecting how individuals find grounding and resilience in times of ecological and societal transformation	Transcendence: reimagining ourselves, our world, and the actions we can take to protect the climate
Resilience and adaptation	Coping responses	The various approaches individuals use to manage their emotional responses and take action	Meaning‐focused Problem focused Emotion‐focused

**TABLE 4:** Most frequently cited definitions of “young people's experiences” of climate change awareness.

Top 9 most frequently referenced “Young people's experiences” of climate change awareness (number of papers)	Example description of term (reference)
**Eco‐anxiety (41)** ^b^ *Convergence*: anxiety about ecological crises	“The concept of eco‐anxiety can include fear of an impending environmental catastrophe, anxiety associated with worsening environmental conditions, anxiety experienced about an ecological crisis, and distress experienced even when there is no immediate physical evidence or proximal physical evidence of climate change or climate‐related threats to oneself” [44]
	“Concern or worry about general ecological crises” [32, p. 2132]
	^a^ **Often cited within papers**: “As a general term for ‘difficult feelings because of the ecological crisis’, eco‐anxiety seems to be quite suitable, because so many forms of these feelings have some characteristics of anxiety” [12, p. 14]
**Emotional responses related to climate change awareness (34)** *Convergence*: wide range of emotions	“…different emotions… such as feeling anxious, feeling or being worried, feeling tense, helpless, powerless, sad, depressed, angry, grieved, guilty, afraid and terrified” [9, p. 101904]
	“The emotional impact of knowing about the climate causes children and young people to experience anxiety, depression, anger, sadness, frustration, grief, and powerlessness” [73, p. 2164]
	^a^ **Often cited within papers**: “…many emotions, including worry, fear, anger, grief, despair, guilt, and shame, as well as hope, although the presence of these vary between individuals” [45]
**Pro‐environmental behaviors (26)** *Convergence*: actions to help the environment	“Pro‐environmental behavior can be defined as trying to act in the best way for the environment” [117, p. 104]
	“Pro‐environmental behavior (PEB) is an umbrella term for the various individual actions taken to reduce negative human impacts on the natural environment” [113, p. 132]
	^a^ **Often cited within papers (many papers cited this author, but interpreted and conveyed their own meanings of the cited work)** [134]
**Climate anxiety (24)** *Convergence*: anxiety about climate change	“The term ‘climate anxiety’ describes how humans perceive, fear and dread the impacts of climate change”[30, p. 123]
	“Climate anxiety is complex, and is recognised to often be based on constructive or practical anxiety. Although painful and distressing, climate anxiety is rational and does not imply mental illness. Anxiety is an emotion that alerts us to danger, which can cause us to search for more information about the situation and find potential solutions”[45]
	“…climate anxiety is defined as concern and worry about climate change” [32, p. 2132]
	^a^ **Often cited within papers**: “…negative emotional consequences associated simply with perceptions of climate change: that is, people's awareness of the problem that is not linked to specific personal experiences…perhaps the most common way of summarising the emotional response is to call it climate anxiety, or more specifically, climate change anxiety” [16]
**Anxiety (18)** *Convergence*: perceived threat‐based arousal and apprehension	“Generally, anxiety can be defined as a state of heightened arousal in response to a perceived threat, often occurring alongside worry or apprehension” [82, p. 4]
	“General anxiety—a negative emotionality characterized by physical symptoms and future‐oriented apprehension where eco‐anxiety focuses on concerns for climate change” [10, p. 3]
	^a^ **Often cited within papers**: None
**Impact on Mental Health (15)** *Convergence*: a state of mental health and wellbeing	“Mental health is a state of mental wellbeing where individuals fulfil their potential, cope with life stressors, and contribute to their community” [119, p. 344]
	“An expansive term, indicating a state of emotional, psychological, and social wellbeing and encompassing our thoughts, feelings, and behaviours” [52, p. 2]
	^a^ **Often cited within papers**: None
**Solastalgia (14)** *Convergence*: pain experienced from loss of home environment	“It describes the pain experienced by the loss or lack of the loved home environment due to environmental degradation, which can be experienced as an attack on the sense of place and endemic place identity” [50, p. 2]
	“…the distress caused by observing changes to an environment that one is connected with” [31, p. 2]
	^a^ **Often cited within papers**: “…solastalgia is the distress that is produced by environmental change impacting on people while they are directly connected to their home environment” [135, p. s95]
**Climate distress (12)** *Convergence*: distress due to climate change awareness	“…the term “climate distress” to cast a wide net across all negative emotional interactions around climate change” [52, p. 3]
	“Psychological distress caused by climate change awareness. This distress can express itself in a wide range of emotions and mental states, comprising general distress, guilt, shame, worry, anxiety, fear, phobia, paralysis, (pre‐) traumatic stress, anger, melancholia, grief, or despair” [92, p. 2]
	^a^ **Often cited within papers**: None
**Depression (9)** *Convergence*: N/A	No definitions provided in the 12 papers that used this term
	^a^ **Often cited within papers**: None

^a^ **Often cited within papers**. Where possible, definitions are taken from the papers with the highest citation counts as well as the definition most often referenced among the other papers in our sample.

^b^ *Convergence*: Given that papers reported multiple definitions for the same terms, we have provided a short description under each term to unify it based on the areas of convergence in the various definitions used in our sample.

#### 3.3.2 Coping Strategies

We identified 52 coping strategies that were reported as being used or as potentially helpful to manage more challenging aspects of the young people's experiences of climate change awareness. According to Lazarus and Folkman, coping involves “cognitive and/or behavioral efforts to manage specific external and/or internal demands” [124, p. 141]. We recognize coping strategies as a specific form of psychological response to the climate crisis. In this review, we initially extracted them separately from other young people's experiences and later included them into categories of young people's experiences seen in Figure 3. These strategies included both adaptive and maladaptive coping strategies. Coping strategies encompass various formats of delivery and contexts, for multiple settings at different levels. For example, delivery modes included strategies or interventions being delivered by a mental health professional or a trained facilitator. This review highlights well‐established and frequently referenced coping approaches, including meaning‐, emotion‐, and problem‐focused coping (Figure 3).

Eight strategies were mentioned in more than five papers included in this review and are summarized in Table S3. Papers often listed multiple strategies, but it was unclear whether these were suggested as being potentially helpful to be used together. Most papers that reported multiple strategies were speculating about their potential value rather than providing data‐based evidence. We also identified an overlap between descriptions of coping strategies and protective factors in the literature, which we aim to explore in future research.

#### 3.3.3 Frameworks, Models, and Theories (Frameworks)

We identified 62 frameworks that have been applied to explain and structure “young people's experiences” of climate change awareness but were not necessarily developed for this purpose originally. Frameworks underpin research and are structured approaches used to guide the investigation and understanding of complex phenomena. Different fields seemed to use certain frameworks. For example, even though the social–ecological framework [125] and Ecological Systems Theory [126] were developed by the same author and use very similar structures, the social–ecological framework seems to be used more frequently by health and behavioral fields, whereas the Ecological Systems Theory has been employed by education and child development fields. Seven of the frameworks were applied in more than two papers (Table 5), with the most frequent being the social–ecological framework (applied five times in the 93 papers).

**TABLE 5:** Most frequently cited frameworks, models, and theories.

Top 7 most frequently cited Frameworks, models, and theories of 62 total identified (number of papers)	Definitions (most highly cited definition)	Example of how the framework, model, or theory was used in the paper
Social–ecological framework (5)	“At the centre, there is an interplay between an individual's genetic and psychological traits, their immediate physical environment, and the relationships and environments that directly surround them. These are further influenced by the broader systems within which they are nested. Applying this framework, it is theorized that climate anxiety results from an interplay of individual factors: the micro‐ (family, peers), *meso*‐ (school, community, local physical environment), *exo*‐ (government, media, global physical environment) and *macro*‐ (culture) systems surrounding children and adolescents.” [30] based on [125]	Crandon et al.’s [30] review drew on a social–ecological framework to discuss how children and adolescents may be uniquely predisposed to climate anxiety
Ecological Systems Theory (4)	“Ecological Systems Theory is a model frequently used in child development studies to describe the inter‐relations of an individual with the system levels they reside within—that is the micro‐ (immediate environment), *exo*‐ (social structures that have influence over micro‐systems), and *macro*‐system (socio‐cultural elements) levels.” [126]	Ma et al.’s. [31] review used Ecological Systems Theory to categorize their findings according to its systems levels. Their review helped to conceptualize what risk factors and protective factors are related to the youth mental health impacts of both direct and indirect exposure to climate change
Developmental psychology perspective (3)	“Developmental psychology helps to explain how young people develop concrete thinking and ideas about time and space, egocentrism, using an evolutionary perspective (humans tend to prioritise short‐term threats and immediate cause‐and‐effect relationships etc.)” [145]	Marinkovic Chavez [76] used the developmental psychology perspective in a review to contextualize the specific vulnerabilities and psychological impacts on children at various developmental stages, providing a conceptual framework to organize and interpret their data on how climate change affects children's mental health
Theory of coping (3)	“Coping is defined as adapting cognition and behaviors to manage specific internal or external demands challenging one's resources. Coping is process‐oriented and dynamic: strategies are actioned in response to a situation, vary depending on context and have secondary effects on an individual's behavior and psychological well‐being.” [124]	Daeninck et al. [86] drew on the Theory of Coping to analyze survey data on coping strategies employed by students to manage climate anxiety
Social Identity Model for Collective Action (2)	“This model argues that collective action is made up of four psychological motivators: moral conviction, emotions, social identity, and perceived group efficacy” [136; 137]	Ruiz‐Dodobara et al. [104] drew on the Social Identity Model for Collective Action to serve as a conceptual framework to analyze a survey looking at whether moral conviction, emotions, social identity, and perceived group efficacy mediate the relationship between social media use and environmental collective action
Transformative education (2)	“An approach to education that goes beyond the transmission of knowledge that learners can remember…” [138] “involves a shift in consciousness that can involve deep learning and changes in behavior, both of which are needed in the context of climate change education.” [139; 140]	Rushton et al. [69] used Transformative education as a conceptual framework to analyze qualitative data on how emotional and psychological support in climate change education can enhance students’ ability to cope with climate anxiety and foster a sense of agency
Climate justice (2)	“Similar to the environmental justice movement which has reframed the environment using social justice as a narrative to catalyze social action, climate justice is a social movement that uses social justice to frame the issue of climate change. Climate justice acknowledges the disproportionate impacts that climate change is having on the world's poorest nations and peoples and also points out that those responsible are the world's wealthiest nations [141], based on [142; 143; 144]	Vamvalis [97] interviewed young activists and educators about their experience of climate justice education, exploring themes in justice‐oriented and decolonial approaches to climate change education and movement building

## 4 Discussion

Our aim was to document the variety of concepts used to describe young people's experiences of climate change awareness, and the conceptual and theoretical frameworks applied to explain and contextualize these phenomena. This review revealed a complex network of ambiguous and often poorly delineated concepts and applied frameworks within the field of climate psychology. We have proposed a categorization that serves as a foundational step towards establishing structure within the field of climate psychology and offers an initial guide to understanding a complex and rapidly growing area of research.

We identified 173 “young people's experiences,” 52 “coping strategies,” and 62 “frameworks.” Our review aligns with the Tapia‐Echanove et al. [127] review which categorized concepts of climate change awareness in youth by affective, behavioral, and cognitive dimensions, of which we used similar categorization in this narrative review. Our review adds to the literature by being inclusive of the full spectrum of young people's experiences such as the affective, behavioral, and cognitive dimensions as well as the subsequent impacts on young people's physical, mental, and social health and well‐being, among others. We developed six categories, with accompanying subcategories, of what we termed “young people's experiences” of climate change awareness. We found 62 frameworks that have been applied to this body of research. Some of the frameworks, such as the developmental and childhood‐focused approaches, are particularly targeted to young people, but most of the other frameworks could also be applied to (older) adult populations, as they are not focused on the developmental stages of young people and children. The 52 coping strategies recognized the well‐cited coping approaches: meaning‐, emotion‐, and problem‐focused coping. The aim of disentangling young people's experiences and coping strategies was not to suggest they are distinct, unrelated, and do not overlap but to create a way to group the concepts into categories and subcategories that helps researchers identify potential connections.

“Young people's experiences” spanned different areas of climate change awareness, including disruptions to social and cultural practices, adverse effects on socioeconomic and political conditions, structural inequalities, and injustices [2, 3, 4]. Understanding young people's experiences in response to climate change awareness is even more important for young people who are transitioning into adulthood marked by brain maturation, social and emotional development, and gaining autonomy over life decisions [25]. This development stage is heightened in the climate crisis, as young people are most affected as well as vocal [4]. Lau et al. [5] call for support of young people to navigate this vulnerable stage of life and support their growth into “future leaders” who are aware of, but not overwhelmed by, global challenges. To fully support young people with their experiences of climate crisis awareness, the field must first map the range of ways in which they are responding and the impacts on their health and quality of life. This review is a step to this goal, which can aid further exploration of the relationships between these young people's experiences.

Eco‐anxiety (*n* = 41) was the most frequently cited “young people's experience” of climate change awareness found in the included papers. Similar to other frequently cited experiences found in this review, such as climate anxiety (*n* = 24) and solastalgia (*n* = 14), eco‐anxiety was inconsistently defined. Eight papers cited Pihkala's [12, p. 14] conceptualization of eco‐anxiety: “as a general term for ‘difficult feelings because of the ecological crisis’, eco‐anxiety seems to be quite suitable, because so many forms of these feelings have some characteristics of anxiety.” Another commonly cited definition was “concern or worry about general ecological crises” [32, p.2132]. The main inconsistency between definitions of “eco‐anxiety” stems from the extent to which it is related to anxiety. Some definitions position eco‐anxiety as an extension of generalized anxiety or as having characteristics of anxiety, whereas some do not mention anxiety at all in the definition and may instead use concepts like “concern or worry,” which muddies the conceptual waters. Another discrepancy with the definitions is related to how it described the relation to “ecological crisis,” as is what is defined in Pihkala's [12, p. 14] and Olsen et al.’s [32, p. 2132] definitions. However, in other definitions, eco‐anxiety is described to be related to “climate change awareness,” “climate crisis,” “environmental doom,” and “worry about the planet.” Again, this inconsistency can create conceptual confusion, as some of these terms are related to the awareness of the climate crisis and others could be considered the exposure of climate events.

Although metrics exist to measure eco‐anxiety (e.g., the Hogg Eco‐anxiety Scale [20]), more consistent and consolidated definitions of eco‐anxiety will help to ensure what is being measured captures the full nature of the evolving understanding of this construct. On the basis of our narrative review, although we do not propose a formal definition of eco‐anxiety, we recommend that any standardized definition should encompass its affective, cognitive, somatic, and behavioral dimensions and make clear whether it is being used to capture emotional experiences outside of “anxiety” or not. Eco‐anxiety should be recognized as a normal and rational response to the climate crisis, existing on a non‐pathological spectrum—though it may reach a tipping point where it adversely affects mental health and well‐being and/or interacts with experiences of mental health disorders and may require clinical support.

In framing our discussion around young people's experiences of climate change awareness, we chose the term “experiences” to reflect the multifaceted and evolving nature of how young people engage with climate change. Although alternatives like “responses” imply a more reactive or outcome‐focused framing, “experiences” allows for a broader and more nuanced understanding—encompassing emotional, cognitive, somatic, behavioral, social, spiritual, and existential dimensions. This framing acknowledges that aspects such as spiritual beliefs, community connections, and overall well‐being can function both as altered outcomes in response to climate awareness and as moderating or mediating factors in how emotions and feelings are experienced and the impact of these. For instance, spiritual or community‐based responses may emerge from climate change awareness, but their strength and quality can also influence whether they serve as protective or risk factors. By using experiences, we aim to capture this dynamic interplay, recognizing that these elements are not static but interact in complex ways that shape young people's awareness and engagement with climate change.

### 4.1 How to Use the Developed Categories From This Review

The subcategories of young people's experience offer a foundational framework for navigating the complex and rapidly evolving field of climate change and mental health. We present these categories as starting points for researchers to explore the diverse dimensions of climate‐related emotional and psychological experiences. They also provide a practical tool for synthesizing existing literature, identifying knowledge gaps, and fostering interdisciplinary collaboration.

### 4.2 Inclusivity in the Climate Psychology Field

High‐income countries still appear to dominate research in this field (66% in this review). However, this evaluation is likely biased because this review only focuses on English‐language literature. However, even the 34% of research found in this review from low‐middle‐income countries is a slight shift upward compared to a similar review in 2021 [2], when almost three‐quarters of papers focusing on climate change and mental health written in English were from high‐income countries. Nevertheless, we found only six studies focusing on the experiences of Indigenous and First Nations young people, highlighting the need for more inclusive research that incorporates different cultural narratives to better understand how experiences and knowledge systems shape young people's experiences in grappling with climate change.

A regional breakdown of literature included in this review is made difficult due to either a lack of specificity or a wide diversity of countries covered in many papers. In the papers that specified location, Europe, North America and Oceania were the most researched, whereas the Caribbean, East, South, and Southeast Asia were the least represented. Although we know that every world region faces specific climate change events (e.g., some regions are more prone to sea‐level rise, whereas some are more prone to bushfire/wildfire), we know little about the interplay between region‐specific climate‐related events and climate change awareness, and how this and other cultural and contextual dimensions shape young people's experiences of climate change awareness in different countries. More research coming from underrepresented regions is urgently needed.

### 4.3 Future of Climate Psychology

The field must critically balance research to develop and refine theoretical and conceptual understandings with evidence to motivate policy and practice investment and inform the development and evaluation of targeted interventions. Most of the climate psychology research so far has focused on developing metrics to measure experiences and identifying coping strategies due to the urgency of the climate crisis. Recent work by Crandon et al. [14] and van Valkengoed and Steg [24] has started to structure the conceptualization of the key constructs, and this review adds an extra understanding of the number of concepts and definitions in this growing field. We hope this review offers a moment of reflection to consider the balance between needing to understand the theoretical basis of the psychology of climate change awareness and taking the much‐needed policy and intervention action, at the same time, due to the urgency of the climate crisis.

The climate psychology field should also invest in understanding the interrelationships between concepts to reduce the risk of conceptual confusion and improve the ways we measure diverse responses to the climate crisis and to understand how to develop interventions based on interacting experiences of climate change. For example, consolidated understandings of how eco‐anxiety is related to key constructs such as climate anxiety, climate agency, and psychological adaptation need to be developed, particularly as different studies often measure relationships between subsets of key constructs, but there remains a key gap in consolidating these studies into a comprehensive framework of established relationships. Papers such as Cosh et al. [128] have started this process, and their review highlighted evidence for relationships between eco‐anxiety and psychological distress, depression symptoms, anxiety symptoms, and stress symptoms but also called for clinical practice assessment to further evidence the relationships. We outline next steps to evidencing interrelationships between concepts in our roadmap below.

To advance the field of climate psychology, it is often recommended to develop standardized definitions and metrics and aggregate concepts and definitions related to young people's experiences such as climate anxiety and eco‐anxiety. Although the standardization of concepts and terminology in climate psychology is a worthwhile goal, our reflections must acknowledge its inherent limitations. Psychological responses to climate change—such as emotions, cognitions, and behaviors—are deeply shaped by culture and other contextual and individual factors. As such, there will always be a degree of specificity in how individuals and communities experience and respond to climate‐related phenomena. For example, Aruta and Guinto [129] reflected that scales developed with Western populations to measure eco‐anxiety and climate‐anxiety only partly capture the experiences of young people in the Global South. When developing standardized definitions and metrics, we need to align with the global movement to decolonize climate psychology and prioritize integrating Indigenous epistemologies, embrace methodological pluralism such as participatory action research, and foster equitable research collaborations [130].

This article represents an initial step toward greater conceptual clarity, but we recognize that consolidation and standardization are complex and evolving processes. Any effort to unify terminology must remain sensitive to the disproportionate impacts of climate hazards on certain communities, as well as the culturally distinct ways in which people adapt, mitigate, and make meaning of climate change and of mental health and well‐being. Perceptions and responses are influenced by factors such as worldview, education, lived experience, and local knowledge systems.

Rather than aiming for rigid uniformity, the field of climate psychology can draw valuable lessons from more established subfields—such as cross‐cultural, Indigenous, and cultural psychology and global mental health—which have long grappled with the balance between conceptual coherence and cultural nuance [131, 132]. These fields remind us that standardization should reduce unnecessary conflation or confusion of concepts but remain necessarily flexible, inclusive, and responsive to the diversity of human experience. With that being said, we have developed a roadmap that can take us on the journey to consolidate concepts and definitions and to understand the interrelationships.

### 4.4 A Roadmap to Unpacking Concepts and Interrelationships

This roadmap offers a guide to unpacking and consolidating the complex and interconnected dimensions of young people's experiences of climate change awareness. Rather than presenting a linear sequence, the roadmap is structured around a series of interrelated phases that can happen simultaneously.

#### 4.4.1 Phase 1: Map Discrete Concepts and Categories Described in the Current Evidence

To fully support young people with their experiences of climate change awareness, the field must first map the range of ways in which young people are responding and the impacts on their health and quality of life. This review has been important to understand how the field is documenting young people's experiences spanning different areas of climate change awareness. Our review has gone beyond the more typical “affective, cognitive and behavioral” responses to climate change awareness and included disruptions to social and cultural practices, adverse effects on socioeconomic and political conditions, structural inequalities, and injustices to encompass young people's holistic experiences of the awareness of the climate crisis [2, 3, 4].

#### 4.4.2 Phase 2: Engage in Interdisciplinary Dialogue

We suggest convening a special working group, inviting the 46 experts from our expert consultation and additional experts, particularly from the Global South and those from and working with Indigenous communities, and those with relevant lived experiences. To define key concepts such as eco‐ and climate‐anxiety, we recommend consensus‐building exercises with the experts, for instance, where they can vote for concept definitions in rounds of Delphi studies and meetings to come to a consensus, or a flexible but consolidated framework of the current understanding of these constructs as they may apply across communities. We recommend bringing in participatory methods that might be suitable for diverse disciplines and lived experience experts, like Talanoa‐style dialogues.

#### 4.4.3 Phase 3: Map Relationships Between Categories/Concepts to Develop a Comprehensive Framework

We recommend that a further comprehensive analysis of the relationship between subsets of key concepts should be conducted, underpinned by a framework, such as the socio–ecological framework [30, 126], adapted with experts to provide a more robust understanding of the relationships. Research projects that collected data on multiple concepts could apply statistical analysis such as structural equation modeling to rigorously understand the relationships between these concepts. This would provide the understanding of relationships between concepts and provide both a bottom‐up approach based on the analysis from the raw dataset combined with a top‐down approach underpinned by a framework.

#### 4.4.4 Phase 4: Machine Learning to Build the Framework

If the field of climate psychology wants to invest in a more comprehensive and robust understanding of the interconnected relationships between young people's experiences and coping strategies, we would suggest using machine learning to build a database that allows researchers to select appropriate framework concepts and metrics from a tailored catalogue of options. This would streamline research efforts and foster collaboration. This is similar to the Human Behavior Change Project [37], which uses AI to systematically analyze and synthesize global evidence on behavior change into an ontology, which is open as an online tool of key concepts that can be searched for. These steps will help to create a more inclusive, comprehensive, and actionable body of knowledge and action in climate psychology.

#### 4.4.5 Phase 5: Risk and Protective Factors

We extracted risk and protective factors from the papers but chose not to include them in this review due to uncertainty about whether they were based on evidence or speculation as many of the papers commented on what they thought could be risk or protective factors without the empirical evidence to back it up. We recommend that future research investigates which factors make young people particularly vulnerable to the negative impacts within the range of experiences of climate change awareness and which factors offer a protective effect, preventing worsening experiences or even making them more positive. This may look like a continuation of Crandon et al.’s [30] model that illustrates the factors that may influence climate anxiety for young people across a range of social–ecological systems.

#### 4.4.6 Phase 6: Testing and Validating Concepts

We suggest testing and validating concepts through empirical studies such as testing metrics and exploring qualitatively to assess the relevance and applicability of concepts across cultures and contexts. The definitions and relationships between concepts may be redefined on the basis of the feedback from the participants and research community as well as identify and resolve inconsistencies in terminology or theoretical approaches.

#### 4.4.7 Phase 7: Dissemination

To ensure efficient dissemination and clear communication of findings, it is important to publish results in accessible formats—such as open access platforms—to promote understanding across various sectors and disciplines. Likewise, targeting interdisciplinary journals can also enhance reach and impact. Incorporating visual representations, such as diagrams and conceptual maps, can further clarify complex relationships between concepts. Additionally, conducting mapping exercises can help illustrate these connections more effectively, fostering deeper comprehension among diverse audiences. A key is disseminating to diverse stakeholder groups, including policymakers, practitioners, and the media, for example.

### 4.5 Strength and Limitations

This review provides a broad and inclusive overview of the climate psychology literature, encompassing a wide range of young people's experiences, coping strategies, and the frameworks used to organize these in the literature to date. This comprehensive approach helps to map the current state of the field and identify key areas for future research. The application of AI and NLP technologies, such as ChatGPT for data synthesis, represents an innovative approach to managing and interpreting large datasets. This method facilitated the categorization and analysis of a vast amount of information on what influences experiences of climate change awareness. Having multiple rounds of human validation, including 46 experts in two expert consultations, provides an extra degree of validation for our interpretations. However, the representation of experts mainly came from the research sector and the Global North reflecting the current state of the literature but also from Australia and the United Kingdom specifically likely due to the nationality of the authors of this article and their networks.

The use of AI in data synthesis presents challenges in transparency and understanding the underlying processes. Although AI tools like ChatGPT can efficiently process large datasets, the “black box” nature of these technologies means that the exact mechanisms by which conclusions are reached are not always clear. This was mitigated by multiple rounds of human validation, including with 46 experts in the field, ensuring that the analysis was informed by diverse perspectives and expertise. Additionally, the review was limited to English‐language papers and concepts, potentially excluding valuable research published in other languages, leading to an incomplete representation of the global climate psychology literature. Taking concepts out of context and the need for human validation may have affected the interpretation of findings, as the reliance on human judgment to validate AI‐generated categories introduces a level of subjectivity. There is also a notable lack of Indigenous knowledge and perspectives from the Global South in the reviewed literature, highlighting the need for more inclusive research that integrates diverse cultural narratives and traditional knowledge systems. This review focused on young people and may not be generalizable to adults.

## 5 Conclusion

In conclusion, this work provides a foundational guide to understanding the expansive and intricate field of climate psychology. By identifying a multitude of “young people's experiences,” “coping strategies,” and “frameworks” from the current literature, the review underscores the complexity and breadth of this rapidly expanding field. The review highlights the growing but still limited inclusivity in research, particularly from low‐middle‐income countries and Indigenous perspectives, and acknowledges the dominance of Western viewpoints and data. Future directions include developing consolidated approaches for aggregating concepts and definitions of “young people's experiences” in response to climate change awareness in young people and exploring interconnected relationships between the metrics, coping strategies, and the frameworks that seek to organize them. We also promote using machine learning to aggregate concepts and understand the interconnected relationships, which would aid the development of a climate psychology framework, showing how the different concepts within climate psychology are linked. Furthermore, this review offers a valuable starting point for further theoretical development and actionable insights in climate psychology. Without concerted action on climate change, rates of mental ill‐health will increase in young people, compromising their overall health and well‐being [5].

## Author Contributions

Emma Louise Lawrance conceptualized the review. Daniella Watson, Emma Louise Lawrance, Ans Vercammen, Fiona Charlson, and John Jamir Benzon Aruta designed the review. Daniella Watson, Tara Crandon, and Teaghan Hogg screened the papers, extracted the data, and conducted the initial analysis of the data. Daniella Watson, Teaghan Hogg, Ans Vercammen, John Jamir Benzon Aruta, and Chloe Watfern facilitated the expert meetings. Fiona Charlson was an expert in an expert meeting. Daniella Watson, Teaghan Hogg, and Tara Crandon lead the synthesis of the data, and all authors contributed to the interpretation. Daniella Watson, Teaghan Hogg, Tara Crandon, and Emma Louise Lawrance drafted and edited the article, and all authors reviewed and approved the final manuscript.

## Conflicts of Interest

The authors declare no conflicts of interest.

## Supporting information



Table S1. Countries that papers focused on high‐, middle‐, and low‐income countries, as defined by the World Bank 2024143.

Table S2. Summary table of included papers.

Table S3. Most frequently referenced coping strategies to manage young people's experiences in response to awareness of the climate crisis.

## Data Availability

Data sharing is not applicable to this article as no datasets were generated or analyzed during the current study.
