# Supplementary material for: Conceptualizing Young People's Experiences of Climate Change Awareness: A Narrative Review
Source: Ann N Y Acad Sci. 2025 Oct 26;1554(1):45–65. doi: 10.1111/nyas.70114 (PMC12728334; doi:10.1111/nyas.70114)
Supplement: Supplementary file 1 — Table S1. Countries that papers focused on high‐, middle‐, and low‐income countries, as defined by the World Bank 2024143. [file NYAS-1554-45-s003.docx]

**Supporting Table S1.**  Countries that papers focused on high-, middle- and low-income countries, as defined by the World Bank 2024^143^

| **Number of studies in Low-Middle Income Countries**  **(n=21 single country studies)** | Brazil (1), China (2), India (1), Indonesia (1), Kenya (1), Lebanon (1), Mexico (2), Peru (1), Philippines (1), Tanzania (1), Turkey (3) |
| --- | --- |
| **Number of studies in High income Countries**  **(n=41 single country studies)** | Australia (12), Canada (6), Italy (3), Germany (5), Finland (1), Hungary (1), New Zealand (1), Portugal (1), Slovenia (1), Sweden (1), United Kingdom (6), United States (3), Indigenous and First Nation communities within high-income countries (6) |
| **Studies that included both low-middle and high income countries (Countries are listed as included in each paper, with each paper numbered separately)** | 1. USA, New Zealand, China, Turkey, and Indonesia  2. Australia, Brazil, Finland, France, India, Nigeria, Philippines, Portugal, the UK, and the USA)  3. USA, Asia, Australia, Europe, Caribbean islands, and Canada  4. Australia, the USA, UK, Canada, Netherlands, Switzerland, India, Finland, New Zealand, and Sweden.  5. Canada, China, Dominica, Indonesia, Philippines and US  6. Australia, Brazil, Finland, France, India, Nigeria, Philippines, Portugal, the UK, and the USA  7. Argentina and Spain |
| **Number of studies which did not specify a country** | 21 |
