# Supplementary material for: Conceptualizing Young People's Experiences of Climate Change Awareness: A Narrative Review
Source: Ann N Y Acad Sci. 2025 Oct 26;1554(1):45–65. doi: 10.1111/nyas.70114 (PMC12728334; doi:10.1111/nyas.70114)
Supplement: Supplementary file 2 — Table S2. Summary table of included papers. [file NYAS-1554-45-s002.docx]

**Supporting Table S2 of included papers**

| **#** | **Author (Year)** | **Country** | **Population** | **Aim** | **Frameworks, models and theories** | **Method** | **Young people’s experiences** | **Coping Strategies** |
| --- | --- | --- | --- | --- | --- | --- | --- | --- |
| 1 | Barchielli et al. (2022)^44^ | Central and Southern Italy | Young adults aged 18-35 | To assess preoccupation and fears on modern issues and the implications for well-being among different age groups. | A climate change risk perception model (CCRPM) | Survey  N = 1831 | Eco-anxiety | Nil |
| 2 | Crandon et al. (2022)^30^ | Non-specific | Young people | To draw on a social–ecological theoretical framework to discuss how children and adolescents may be uniquely predisposed to climate anxiety | Social–ecological framework | Review | Climate anxiety | Multiple strategies, community participation, meaning-focused coping, problem-focused coping, nature-based solutions, youth empowerment and policy advocacy, science communication and education |
| 3 | Hickman et al. (2021)^45^ | Australia, Brazil, Finland, France, India, Nigeria, Philippines, Portugal, the UK, and the USA | Young people aged 16-25 | To better understand the feelings, thoughts, and functional impacts associated with climate change awareness among young people globally. | Nil | Survey  N = 10,000 | Climate anxiety  Beliefs about government | Spaces for emotional expression |
| 4 | Jones & Davison (2021)^46^ | Tasmania, Australia | Young people in university aged 18-24 | To investigate the ongoing and formative significance of emotional experiences of climate change education in childhood. | Nil | Interviews  N = 21 | Feelings on the generation gap, powerlessness, beliefs about government | Nil |
| 5 | Clayton & Karaszia (2020)^16^ | United States | About 50% between the ages of 25 and 34, but ranged from the category 18–24 to three people who were 75 or older. | To develop a measure of climate change anxiety that would allow for consistency in measurement and understanding. | Nil | Surveys  N = 217 | Climate anxiety | Nil |
| 6 | Olsen et al. (2024)^32^ | Australia, New Zealand, Finland, United States of America, England, Norway, Ireland, Singapore, Sweden | Students’ and/or educators | To answer the call from Pihkala’s (2020) previous review for more concrete information on educational approaches to support learners in processing eco/climate anxiety. | Transformative education  Transformative Sustainability Learning | Review  articles = 15 | Eco anxiety, climate anxiety | Youth empowerment and policy advocacy |
| 7 | Breen et al. (2024)^47^ | United States, New Zealand, China, Turkey, and Indonesia | College students enrolled at community colleges and in post-graduate programs | To understand current knowledge focusing on environmental disasters and college students in order to determine future research needs. | Nil | Review articles = 67 | Stress and anxiety, depression, PTSD, suicidality | Help-seeking behaviours |
| 8 | Proulx et al. (2024)^48^ | Non-specific | Children and adolescents | To explore climate change effects across the six domains of health and well-being outlined in this comprehensive agenda, including physical and mental health, responsive relations and connectedness, nutrition, safety and security, and learning opportunities. | Nil | Review | PTSD, eco anxiety, distress, financial strain | Responsive caregiving |
| 9 | Voltmer & von Salisch (2024)^49^ | Non-specific | Children and young people | To synthesize the literature on how subjective well-being and a sustainable lifestyle are linked with the personal psychological resources of young people, both theoretically and empirically, and to sketch some interventions that promote both subjective well-being and pro-environmental values, intentions, and behaviour. | Pleasure-Goal Regulation-Meaning Theory of Well-Being | Review | Climate distress, subjective well-being, pro-environmental behaviours | Meaning-  focused coping, mindfulness |
| 10 | Tsevreni et al. (2023)^50^ | Non-specific | Children and young people born between 1997–2012 | To analyze the eco-anxiety and climate anxiety aspects of Generation Z, based on a critical review of studies on children’s and young people’s ecological feelings worldwide, alongside a study of actual data on natural disasters per country since the year 2000. | The Existential Dimension of Eco-Anxiety | Review  articles = 22 | Climate anxiety, solastalgia, environmental melancholia | Nil |
| 11 | Treble, Cosma & Martin (2023)^51^ | Non-specific | Children and adolescents | To conduct a narrative review on research surrounding children’s and adolescents’ experiences of emotional and mental health and wellbeing in relation to climate change. | Nil | Review | Eco anxiety, climate anxiety | Meaning-  focused coping |
| 12 | Martin et al. (2023)^6^ | Non-specific | Young people aged 10–24 | To increase the understanding about this, survey instruments are needed that measure the negative emotions young people experience about climate change. | Nil | Review  articles = 43 | Environmental beliefs, pro-environmental behaviour | Meaning-  focused coping |
| 13 | Koder, Dunk & Rhodes (2023)^52^ | Australia, United States, United Kingdom, Canada, Netherlands, Switzerland, India, Finland, New Zealand, and Sweden | Children, students, adults, and elderly. | To explore how clinicians respond to and feel about climate change distress in therapy, identify interventions to address climate distress, inform research and contribute to the knowledge on addressing climate distress. | Ecopsychology, Climate psychology, Big 5 Model of Personality, Norm-activation theory | Review  N = 23,163  articles = 44 | Mental health, eco anxiety, maladaptive responses, climate denial, climate distress, fear of world ending, pro-environmental behaviour, climate action, awareness, beliefs about government, concerns about future | Therapy, nature-based solutions, community participation, Place- and legacy-based interventions |
| 14 | Cianconi et al. (2023)^53^ | Non-specific | Urban populations, with a section on young people | To contribute to the understanding of the effects that climate change has on the mental health of the urban population. | Nil | Review | Climate chaos, heat island effect | Nature-based solutions |
| 15 | Walinski et al. (2023)^54^ | Non-specific | Human populations - no age restriction | To summarize the current global evidence regarding the effects of climate change on mental health. | DSM-V | Review  articles = 128 | PTSD, anxiety, general psychiatric outcomes, food insecurity, forced permanent displacement | Nil |
| 16 | Ramadan et al. (2023)^55^ | Non-specific | Young people, teens, students, adolescents, children, or similar | To search the literature for original research investigating mental health and climate-related negative emotions in young people. | Nil | Review articles = 26 | Climate anxiety, climate worry, eco-anxiety, ecological grief, ecological stress, eco-paralysis, environmental concerns, environmental distress, environmental grief, solastalgia, betrayal, pro-environmental behaviour, beliefs about government, perceived financial security | Meaning-  focused coping |
| 17 | Jylha et al. (2023)^56^ | Non-specific | Adults and youth | To understand what leads people to deny even the strongest evidence and distrust the scientific method, with the main focus on climate change denial. | System justification theory, social dominance orientation (SDO), right-wing authoritarianism (RWA) | Review | Science denial, anger, conservatism | Science communication and education,  Motivated reasoning |
| 18 | Boluda-Verdu et al. (2022)^9^ | Non-specific | General populations | To critically evaluate the evidence on eco-anxiety related to climate change and its health implications in general populations. | Nil | Review articles = 12 | Eco anxiety, emotional responses, pro-environmental behaviour, concerns about the future, | Nil |
| 19 | Leger-Goodes et al. (2022)^57^ | Non-specific, but English and French language included | People below the age of 18 | To identify the available evidence on the topic of eco-anxiety in children, clarify mental health consequences brought by the awareness of climate change in this population, and identify knowledge gaps in the literature and considerations for future research. | Nil | Review articles = 18 | Eco anxiety, ecophobia, emotional responses, concerns about the future, anticipating a significant loss of biodiversity | Meaning-focused coping, problem-focused coping, emotion-focused coping |
| 20 | Singh, Xue & Poukhovski-Sheremetyev (2022)^58^ | Non-specific | Targeting health professionals | To offer the beginnings of a framework for action in the context of climate change and youth mental health, before calling on the profession to re-examine its role - and its very purpose - to better address the climate crisis. | Social lens | Opinion piece | Eco anxiety, solastalgia | Nil |
| 21 | Ma, Moore & Cleary (2022)^31^ | United States, Asia, Australia, Europe, Caribbean islands, and Canada | Young people aged between 0-24 | To scope the current research on what and how risk factors and protective factors are related to the mental health impacts of both direct and indirect exposure to climate change for young people. | Ecological systems theory | Review articles = 92 | Climate anxiety, eco anxiety, solastalgia, ecological grief,, pro-environmental behaviour, depression | Avoidance and escape-oriented coping strategies, problem-focused coping, |
| 22 | van Nieuwenhuizen et al. (2021)^59^ | Non-specific | Non-specific | To review recent literature on the effects of climate change on child and adolescent mental health and discuss treatment and engagement by clinicians. | Social determinant of child and adolescent mental health, ecological determinants of mental health, social cognitive theory, | Review | Posttraumatic stress, emotional responses, aggression, changes to sleeping habits, changes to eating habits, pro-environmental behaviour, dreams, increase in emergency room visits, suicide rates, psychiatric medications, financial strain | Meaning-focused coping, civic engagement, activism, meaning and problem-focused coping, Trauma-Focused Cognitive Behavioral Therapy |
| 23 | Pereira & Freire (2021)^60^ | Mainly in North America and Europe | Studies relating to adolescents and young people aged 10-24 | To emphasize that a developmental perspective is fundamental within the interdisciplinary studies concerning climate change. Specifically, we focus our research on how the Positive Youth Development framework may inform future approaches to promote adolescents' and young adults' well-being and engagement in the context of climate change. | Developmental perspective on climate change, bioecological systems perspective, positive youth development framework | Review articles = 13 | Agency, hope, locus of control, behavioural intention, pro-environmental behaviour, environmental engagement | Nature-based solutions, meaning-focused coping |
| 24 | Bessaha et al. (2022)^61^ | Canada, China, Dominica, Indonesia, Philippines and United States | Youth and young adults aged 15–29 | To review the literature on disaster mitigation, response, and recovery following natural disasters with a focus on the engagement of youth and young adults. | Disaster Risk Reduction (DRR), Youth engagement and participation | Review articles = 19 | Resilience | Community participation, youth empowerment and policy advocacy, education and community partnership, post-disaster rescue efforts |
| 26 | Ramadan & Ataallah (2021)^62^ | Non-specific | Non-specific | To understand the impact of climate change-related disasters on mental health and the different methods of solving the problem at the corporate level, by trying to decrease greenhouse gas emissions to zero, and at the individual level by learning how to cope with the impacts of those disasters. | Nil | Review | Solastalgia, eco anxiety, eco-paralysis, emotional responses, PTSD, posttraumatic stress, aggression, criminal behaviour, neurodevelopmental and behavioural disorders, abuse (child and domestic), loss of livelihoods, forced permanent displacement, impact on community well-being, financial damage | List of structural strategies |
| 26 | Benoit, Thomas & Martin (2022)^63^ | United States | Young people | To analyze the discourse in major American newspapers about children and their parents during the evolving climate crisis, with specific interest in how the lay press addressed: (a) the feelings and actions of young people; and (b) the expectations of parents during the climate crisis. | Childism, existential psychology, Maslow's self-actualization | Review articles = 133 | Anxiety, earth emotions, solastalgia, eco-anxiety, ecological grief, cognitive dissonance, death anxiety, existential guilt, normalcy, concerns about the future, transcendence, self-actualisation, adultified youngsters, innocent victims | Parents are expected to tame children’s anxieties by focusing on action |
| 27 | Vergunst & Berry (2022)^64^ | Non-specific | Prenatal up to adolescence | To describe how a developmental perspective can inform and structure current thinking around the effects of climate change on psychological health and well-being from the perinatal period to adulthood. Also, to advance thinking on how these threats can be conceptualized, measured, and prioritized by providing an initial framework that can guide research, policy development, and intervention planning. | Developmental psychology, ecological systems theory | Review | Solastalgia, eco anxiety, anxiety | Community participation, science communication and education |
| 28 | Gislason, Kennedy & Witham (2021)^65^ | Mainly Canada, but also Australia, United States, United Kingdom, Netherlands, Sweden, Portugal, and Finland | Children and youth | To gain a deeper understanding of the relationship between climate change and children and youth’s mental health. | Intergenerational climate justice approach, Ecological Determinants of Health (EDoH), Social Determinants of Health (SDoH), eco-social approach, social inequities in health approach, Whole person, whole community, whole of life, and whole of planet approach, Framework for Collaborative Action on Health and Climate Change | Review articles = 58 | Ecological grief, fear of the world ending, political engagement, climate action, concerns about the future, lack of control | Resilience, strength-based community and participatory approaches, strengths- and asset-based interventions and frameworks, Cognitive Behavioral Therapy (CBT) |
| 29 | Clemens, von Hirschhausen & Fegert (2022)^66^ | Mainly Europe | Children and adolescents | To provide an overview of potential mental health consequences of climate change in children and adolescents. | Nil | Review | Posttraumatic stress, emotional responses, behavioural disorders, logical thinking, hospital admissions, food insecurity, forced permanent displacement | Nature-based solutions, meaning-focused coping, self-efficacy |
| 30 | Rother et al. (2022)^43^ | Sub-saharan africa | Children (newborn to less than 10 years) and adolescents (10-19 years) | To examine research findings on the direct and indirect impacts of EWE on the mental health of children and adolescents living in SSA to inform protective adaptation strategies and promote resilience. | Children's Climate Risk Index (CRI) | Review articles = 2 | Psychological distress | Structural resources (camps that housed displaced families),  Social support |
| 31 | Clayton et al. (2023)^67^ | Australia, Brazil, Finland, France, India, Nigeria, Philippines, Portugal, United Kingdom, and United States | Young people aged 16–25 | To explore responses to climate change and associated gender differences in a young sample than most previous research has used. | Social–ecological framework | Survey  N = 10,000 | Climate anxiety | Nil |
| 32 | Jones & Lucas (2023)^68^ | Australia | Young people aged 15-19 | To understand what emotions young people associate with climate change, who they talk to about these emotions, what influences whether they talk or about these emotions, and how they feel when they talk to others about climate change. | Post-dualist, affect theory, | Survey  N = 1943 | Eco anxiety, coping, relationships and connectedness | Nil |
| 33 | Rushton et al. (2023)^69^ | England | Young people aged 11–18 | To consider how secondary school pupils from a diverse range of geographical and socio-economic contexts in England experience and understand climate change. | Transformative education | Focus groups  N = 85 | Nil | Nil |
| 34 | Russell (2024)^70^ | Australia | Young people aged 18-24 | To generate inductive insights linking emotional experiences of climate change in the context of different systemic influences, and reveal any patterns, similarities or differences in experiences between participants. | Social–ecological framework, ecological systems theory | Interviews  N = 14 | Climate distress, eco anger, climate grief, solastalgia, feelings of uncertainty, agency, beliefs about government, concern about the future, | Nil |
| 35 | Ojala (2023)^71^ | Non specific | Young people - mid childhood to early adulthood | To present an overview of research about young people and climate change concerning climate change and mental well-being, coping with climate change, private-sphere pro-environmental behaviour as a form of pro-social development, and climate change and political socialization. | Nil | Review | Mental wellbeing, climate change anxiety, political engagement, climate action, concerns about the future | Meaning-focused coping, emotional regulation |
| 36 | Newberry Le Vay et al. (2024)^72^ | United Kingdom | Children and young people | To explore why promoting good mental health and wellbeing and building psychological resilience can help achieve climate change education outcomes, and why not doing so risks harming children and young people’s mental health. Also, to explore how integrating discussions about emotions, mental health, and coping strategies within climate change education can be a route into wider discussions about mental health, to support children and young people in the context of rising mental health needs. | The whole school approach | Review | Mental wellbeing, climate distress, environmental knowledge | Science communication and education, youth empowerment and policy advocacy |
| 37 | Ediz & Yanik (2023)^73^ | Turkey | Young people aged 15 to 24, including climate activists and those who are not | To determine the impact of climate change awareness among Turkish youth (both climate activists and nonactivists) on their mental health (climate anxiety and hopelessness) and provide an overall perspective. Also, to explain and clarify the terminology related to the relationship between youth, climate change, activism and mental health. Lastly, to define the scope for future research areas and provide recommendations. | Nil | Survey  N = 306 | Climate anxiety, emotional responses, mental ill-health, climate change related mental health impairments, stress, powerlessness, activism, awareness, concerns about the future | Problem focused-coping |
| 38 | Teo et al. (2024)^74^ | Australia | Young people aged 15-19 | To evaluate the extent of climate change concern in young people aged 15–19, its association with various demographic factors and its impact on psychological distress and future outlook. | Nil | Survey  N = 18800 | Emotional responses, psychological distress, climate concerns | Nil |
| 39 | Yatirajula et al. (2023)^75^ | Faridabad and Hyderabad, India | Adolescents and young people aged 16-24 | To understand responses to both climate and COVID-19 crises. Also, to understand the concerns and desires for the future that the study participants wanted to see “built back” from the COVID-19 crisis. Lastly, to understand the sense of agency in relation to the COVID-19 and climate crises | Nil | Survey  Survey  N = 536 | climate change related mental health impairments, climate change hopelessness | Problem-focused coping, meaning-focused coping |
| 40 | Chavez et al. (2024)^76^ | Victoria, Australia | Young people aged 12-25, living in Victoria and interested in climate change | To understand the experiences of a group of young people from Victoria, Australia, a region affected by the increasing frequency and severity of climate-change-induced disasters, such as the 2019–2020 Black Summer bushfires and 2021–2022 floods. | Developmental psychology, ecological systems theory, Strengths-based, holistic framework | Participatory research N = 31 | Emotional responses, solastalgia, eco-anxiety, “capitals” (i.e., strengths, resources), powerlessness, feelings about the generation gap, pro-environmental behaviour, climate action, awareness, desire for systemic change, concerns about the future, | Avoidance and escape-oriented coping strategies, nature-based solutions, community participation |
| 41 | Patrick et al. (2023)^77^ | Australia | Professionals working with young Australians aged 18–24 | To understand the drivers of climate-related mental well-being among young people, what actions could be taken to promote climate-related mental wellbeing, and what value a systems approach can bring to exploring climate-related mental wellbeing among young people. | Systems thinking | Group Model Building (GMB)  N = 14 | Emotional responses, eco anxiety, ecological grief, solastalgia, powerlessness, beliefs about Government | Community participation, youth empowerment and policy advocacy |
| 42 | Martin, Roswell & Cosma (2024)^78^ | Austria, Belgium, Switzerland, Czechia, Germany, Estonia, Spain, Finland, France, United Kingdom, Hungary, Ireland, Israel, Iceland, Italy, Lithuania, Netherlands, Norway, Poland, Portugal, Russian Federation, Sweden, Slovenia | Young people aged 15-35 years | To explore whether there is an association between mental wellbeing (happiness and life satisfaction) and worry about climate change as well as belief about personal responsibility. Also, to explore whether the above relationships are moderated by age, gender and frequency of climate change thoughts. Lastly, to investigate whether belief about personal responsibility moderate the relationships between mental wellbeing and worry about climate change. | Nil | Survey  N = 12,117 | Mental wellbeing, subjective wellbeing, climate worry, happiness/life satisfaction, personal responsibility | Nil |
| 43 | Boyd et al., (2024)^79^ | Port Macquarie, Australia | Regional youth aged 12-25 | To address limited research on eco-anxiety in regional youth by focussing on the lived experiences of regional Australian youth with recent experience of climate related disasters alongside clinical insights from those involved in their care. | Nil | Focus Groups  N = 25 (13 clinicians, 12 youths) | Emotional responses, feelings about the generation gap, beliefs about government | Multiple strategies, Avoidance and escape-oriented coping strategies, Meditation, Activism, Creativity, Mindfulness |
| 44 | Wang & Liu (2024)^39^ | Nonspecific | Students (nonspecific age) | To discuss how air pollution affects students' mental health and to recommend joint efforts to help students cope with the constantly changing environment and maintain a good level of mental health, and more effectively address the challenges of global climate change and air pollution and promote the achievement of the United Nations Sustainable Development Goals. | Biophilia hypothesis | Editorial/  Opinion piece | Emotional responses, feelings about the generation gap, stress, cognitive function | Multiple strategies  Physical exercise, Normal social activities |
| 45 | Mat et al. (2024)^80^ | Istanbul, Turkey | Nursing students | To shed light on the perceptions of future health professionals about climate change by revealing nursing students’ worry and hope levels about climate change. | Nil | Survey  N = 260 | Emotional responses, eco anxiety, mental ill-health, mental health, climate worry, hope, environmental knowledge | Nil |
| 46 | Er et al. (2024)^81^ | Istanbul, Turkey | Nursing students over the age of 18 | To determine the eco-anxiety levels of nursing students and the relationship between eco-anxiety and mental health. | Nil | Survey  N = 609 | Eco anxiety, emotional responses, climate change related mental health impairments, climate change hopelessness, list of emotions, powerlessness, pro-environmental behaviour, activism, awareness, outcome efficacy | Problem focused coping |
| 47 | Mateer (2024)^82^ | Nonspecific | Teachers and young people (age nonspecific) | To explore how to educate young people on climate change and respect their autonomy while also mitigating the potentially negative psychological effects of this learning process. | Social connection model, role-ideal model | Opinion piece | Anxiety, climate anxiety, psychological distress, maladaptive responses, adaptive or maladaptive eco-anxiety, agency, empowerment, climate action | Science communication and education, strength-based community and participatory approaches, youth empowerment and policy advocacy, list of structural strategies |
| 48 | Lykins et al. (2023)^40^ | New South Wales, Australia | Young people between the ages of 16-25 | To assess mental health and climate change concern in young people following the Black Summer bushfires in Australia. | Nil | Survey  N = 746 | Eco anxiety, climate concerns, climate distress, emotional responses, psychological distress, risk perceptions | Nil |
| 49 | Kaligis et al. (2023)^83^ | Indonesia | Young people/adolescents aged 10–19 | To shed light on issues (of climate change impacts on youth mental health) to deepen the understanding of the complexities surrounding adolescent mental well-being and its relationship with climate change. | Nil | Opinion piece | Eco anxiety, climate change related mental health impairments, emotional responses, climate concerns, list of emotions, resilience, climate action | Community participation, youth empowerment and policy advocacy |
| 50 | Patrick et al. (2023)^84^ | Australia | Individuals aged over 18 | To understand the impact of climate change on mental health in the Australian population and identify populations who are most at risk of climate-related mental health burden. | Nil | Survey  N = 5483 | Eco anxiety, pre-traumatic stress, anticipatory traumatic stress, psychoterratic syndromes, mental health, posttraumatic stress, functional impairment, cognitive function, risk perceptions, climate change beliefs | Avoidance and escape-oriented coping strategies |
| 51 | Prencipe et al. (2023)^85^ | Tanzania | Youth aged 18–23 | To measure self-perceived climate change distress among young people living in rural, low-resource communities of Tanzania, and to identify whether climate-sensitive risk factors and climate distress were associated with worse mental health in this population. | Nil | Survey  N = 2053 | Emotional responses, eco anxiety, solastalgia, ecological grief, climate anxiety, climate distress, climate change related mental health impairments, stress, emergent and general psychiatric outcomes, awareness, health-related quality of life | Religious values |
| 52 | Leger-Goodes et al. (2023)^57^ | Quebec, Canada | Children aged 8–12 | To gain insight into the ways in which children experience eco-anxiety using qualitative methodologies and semi-structured interviews. We also aimed to capture parents’ awareness of their children’s concerns through a questionnaire. | Theory of coping, emotion classification | Interviews  N = 12 families, with 12 parents and 15 children | Emotional responses, eco anxiety, powerlessness, feelings about the generation gap, fear of the world ending, list of emotions, pro- environmental behaviour, environmental knowledge, concerns about the future | Problem focused-coping, emotion-focused coping, meaning-focused coping, places for emotional expression, science communication and education |
| 53 | Daeninck, Kioupi, Vercammen et al. (2023)^86^ | United Kingdom | Students at a higher education institution (age nonspecific) | To understand whether climate anxiety would be raised among environmental degree students, to explore the variation in the use of coping strategies, and to examine future planning in relation to climate concerns among students. Also, to investigate the effect of degree focus (environmental vs. non-environmental) and climate anxiety on decision-making around various life events. | Theory of coping | Survey  N = 473 | Emotional responses, ecological grief, ecological stress, environmental distress, climate change distress, eco-angst, climate change worry, climate anxiety, eco-anxiety, and solastalgia, environmental beliefs, personal responsibility, efficacy, feelings about the generation gap, climate action, future planning, financial decisions | Problem focused-coping, meaning-focused coping |
| 54 | Chou et al. (2023)^87^ | Brazil: the cities of São Paulo (state of São Paulo) and Salvador (state of Bahia), as well as several villages of Ilha de Itaparica (state of Bahia) | Adolescents aged 5 to 18 | To better understand children’s and adolescents’ experiences of climate change in Brazil, a middle-income country marked by social and economic inequalities. Also, to elucidate more effective forms of climate-change communication to help young people move from fear and paralysis to hope and action. | The psychological climate paradox, developmental psychology | Interviews  N = 50 | Emotional responses, death anxiety, list of emotions, resilience, pro- environmental behaviours, changes to sleeping habits, awareness | Storytelling, community participation, science communication and education, youth empowerment and policy advocacy |
| 55 | Abebe, Bailey & Radu (2023)^88^ | Canada | University students (age nonspecific) | To describe the creation of an educational intervention for nursing students, as part of ongoing professional development for mental health nurses. Also, to advance the position that arts-based approaches allow people to engage with the anxieties and strong emotional reactions to challenges such as climate change while maintaining motivation to make positive behavioural changes and reducing hopelessness and inaction. | Transtheoretical model of change | Educational project | Eco anxiety, eco paralysis, ecological grief, pro- environmental behaviours | Youth empowerment and policy advocacy, community technology |
| 56 | Smith et al. (2023)^89^ | British Columbia, Canada | Young people aged 18 to 25, nulliparous, assigned female at birth | To explore how climate change impacts the pregnancy intentions of young women in Canada and their perspectives towards childbearing. | Nil | Auto-photography and interviews  N = 7 | Eco anxiety, loss, feelings on the generation gap, concerns about the future, desire for systemic change | Nil |
| 57 | Mebane et al. (2023)^90^ | Italy | High school students (mean age 16.12 years) | To propose a new psychological environmental intervention program seeking to increase students’ awareness of climate change and related emotions and to promote students’ empowerment. | Nil | Pilot intervention  N = 25 | Emotional responses, empowerment, pro- environmental behaviours | Science communication and education |
| 58 | Lass-Hennemann (2023)^91^ | Saarland, which is a federal state of Germany | 7 to 9th graders of all secondary schools (the majority between 12–16 years old) | To assess the impact of climate change, COVID-19 and the Russia-Ukraine War on mental health (depression, anxiety, and health-related quality of life) in secondary school students. Also, to assess known predictors of mental health, such as socio-economic factors, individual life stressors, and resilience factors (self-efficacy, expressive flexibility) as covariates. | Nil | Survey  N = 3,998 | Fearful, anxiety, depression, self efficacy, health related quality of life | Expressive flexibility |
| 59 | Gebhardt et al. (2023)^92^ | Heidelberg, Germany | Patients of a psychosomatic outpatient clinic | To explore whether persons with pre-existing mental health diagnoses show a quantifiable amount of psychopathology due to psychoterratic syndromes through climate change awareness (PSYCA). Also to explore whether psychopathological symptoms of depression, anxiety, and post-traumatic stress predict psychopathological PSYCA symptoms in persons with pre-existing mental health diagnoses. Lastly, to see if personality factors or demographical factors predict psychopathological PSYCA symptoms in persons with pre-existing mental health diagnoses. | Nil | Survey  N = 89 | Climate distress, climate change related mental health impairments, psychoterratic syndromes, anxiety, depression, PTSD | Nil |
| 60 | Wu et al. (2023)^93^ | Canada | Students aged 15-17 | To assess levels of climate concern among Canadian adolescents using the Youth Development Instrument (YDI), and to collaborate with adolescents to adapt an existing climate anxiety scale to be included in the YDI survey. | Nil | Survey  N = 2,306 | Climate anxiety, eco anxiety, climate concerns, mental health | Nil |
| 61 | Marks et al., (2023)^94^ | South West of the United Kingdom | Students aged 16-18 years | To explore how a schools-based, co-created workshop for school pupils could use a community-oriented space to explore their eco-emotions, address feelings of isolation and engender a sense of realistic, active hope, using storytelling and images of possible futures. | Nil | Pilot Intervention  N = 4 | Climate anxiety, eco anxiety, climate grief, solastalgia, ecological distress, emotional responses, climate distress, concerns about the future | Constructive hope, spaces for emotional expression, storytelling |
| 62 | Shao & Yu (2023)^95^ | China | Chinese college students (mean age 21.08) | To verify the causal relationship between media coverage, eco-anxiety, and pro-environmental behavior, and examine the moderating effect of resilience. | The risk amplification framework | Experiment (N = 284) and survey (N = 507) | Eco anxiety, eco-paralysis, adaptive or maladaptive eco-anxiety, resilience, pro-environmental behaviour | Problem-focused coping |
| 63 | Sampaio et al. (2023)^96^ | Portugal | University students aged 18-25 | To validate the Portuguese version of the Hogg Eco-Anxiety Scale (HEAS) in young adults and examine the associations among eco-anxiety, sociodemographic characteristics, and pro-environmental behaviours | Nil | Survey (623), follow up (200) | Eco anxiety, climate anxiety, eco-paralysis, emotional responses, pro-environmental behaviour | Nil |
| 64 | Vamvalis (2023)^97^ | Canada | Elementary and secondary educators, and youth climate justice activists aged 16–20 | To explore possible educational responses that recognize the embodied consequences of climate injustice and inaction on youth mental health and well-being. | Climate justice | Semi-structured interviews and focus groups  6 educators, 3 youths | Climate anxiety, climate dread, climate grief, climate change related mental health impairments, climate despair, environmental stress, environmental anxiety, hope, activism, agency | Nil |
| 65 | Plohl et al. (2023)^98^ | Slovenia | Young people (aged 18-24) | To validate Slovenian versions of the Climate Anxiety Scale (CAS) and the Climate Change Worry Scale (CCWS) among Slovenian youth. | Nil | Survey  N = 442 | Psychological distress, insomnia, suicidality, eco anxiety, climate anxiety, climate worry, anxiety, stress, resilience, support for climate policies, pro-environmental behaviour, risk perceptions, neuroticism | Nil |
| 66 | Ramirez-Lopez et al. (2023)^99^ | Mexico | Undergraduate university students (aged 18-25) | To evaluate the relationship between climate anxiety and psychosocial predictors of vulnerability in a sample of Mexican college students. | Nil | Survey  N = 461 | Mental health, eco anxiety, climate anxiety, anxiety, emotional responses, pro-environmental behaviour, awareness, prosociality | Nil |
| 67 | Finnegan (2023)^100^ | England, United Kingdom | Sixth form students (aged 16-18) and secondary teachers | To explore reported climate education practices and future outlooks through a questionnaire completed by 16–18-year-old secondary school students and teachers. | Nil | Survey  512 students, 69 teachers | Climate anxiety, climate hope, action competence | Nil |
| 68 | Wullenkord & Ojala (2023)^101^ | Sweden | Swedish high-school students (mean age 17.17) | To shed light on the development of climate worry as climate change progresses and to narrow the research gap on young people's climate worry, we investigate relations between different forms of climate worry, coping with this worry, and relations to well-being, climate pessimism, and pro-environmental behavioral outcomes. | Transactional theory of coping | Survey Study 1 (N = 321) and Study 2 (N = 474) | Climate worry, climate anxiety, emotional responses, climate concerns, mental wellbeing, subjective wellbeing, pro-environmental behaviour | Meaning-focused coping, problem-focused coping, emotion-focused coping |
| 69 | Macdonald et al. (2023)^41^ | Victoria, Australia | Young people aged 14–25 | To demonstrate how a youth-focused intervention enabled young people to become agents of change in bushfire recovery. | Nil | Case study | Mental health, emotional responses, acts of citizenship, desire to engage in disaster recovery and preparedness | Nil |
| 70 | Murray et al. (2023)^42^ | Aotearoa New Zealand | Informants from organisations that provided ocean or coastal literacy education to school-aged children (primary school (age 5–12), high school (ages 13–19) or both) | To investigate how ocean literacy educators in Aotearoa New Zealand view the content they deliver in relation to the emotional wellbeing of young people. | Nil | Semi-structured interviews  N = 21 | Mental ill-health, anxiety, grief, depression, climate anxiety, holistic wellbeing, positive and negative emotions, loss of meaning, concerns about the future | Nil |
| 71 | Poortinga, Demski & Steentjes (2023)^102^ | United Kingdom | Post-War generation, the first half of the baby boomer generation, the second half of the baby boomer generation, Generation X, Millennials, and Generation Z | To examine generational differences across different types of climate engagement including cognitive and affective dimensions. | Nil | Survey  1st wave (N = 1893 ), 2nd wave (N = 1001), 3rd wave (N = 1087) | Climate concerns, climate anxiety, emotional responses, climate fear, climate change beliefs, risk perceptions | Nil |
| 72 | Oberauer et al. (2023)^103^ | Southern Germany | Teenage secondary school students | To address critical gaps in climate change education research with regard to emotions triggered in teenage students learning about climate change, the students’ complexity thinking competence in the context of climate change consequences, and the interconnections between different types of emotions and the levels of complexity thinking competence in teenagers’ explanations of climate change. | Nil | Intervention  N = 356 and N = 246 | Emotional responses, critical emotional competence, climate action, competence of complexity thinking | Nil |
| 73 | Ruiz-Dodobara et al. (2023)^104^ | Lima, Peru | University students (aged 18 to 35) | To analyze whether Social Identity Model for Collective Action (SIMCA) variables mediate the relationship between social media use and environmental collective action. | Social identity model for collective action | Survey  N = 259 | Anger, climate action, moral conviction | Nil |
| 74 | Ettinger et al. (2023)^105^ | International (40 different countries) | Students from the University of Oxford, UK | To discuss experiences designing the Talk Climate Change campaign and reflect on the lessons its outcomes hold for climate change communication. | Dialogue-driven approach, spiral of silence theory | Written dialogue  N = 1000 | Climate anxiety, emotional responses, feelings of uncertainty, pro-environmental behaviour, pluralistic ignorance, concerns about the future | Climate conversations, spaces for emotional expression, values |
| 75 | Zurba et al. (2024)^106^ | Canada | Youth mostly aged between 15-25 | To provide insights on the ways that youth express and process the emotions arising from their involvement in climate action. Specifically, to understand the ways youth come to know, conceptualize and reflect on climate change as well as how youth’s emotions in response to climate change impacts their everyday lives. Also, to determine the modalities in which youth are expressing and processing such emotions. | Nil | Photovoice interviews  N = 15 | Holistic wellbeing, emotional responses, mental ill-health, climate grief, ecological grief, solastalgia, eco anxiety, emotions and impacts on everyday life, positive and negative emotions, climate action, concerns about the future, personal responsibility, beliefs about government | Escape, perception of nature's resilience, multiple strategies, Education and training, use of humour, Emotional regulation, Art, Families provided inspiration for many actions taken by youth, Talking and communicating with similar-minded people |
| 76 | Lucas et al. (2024)^107^ | Tasmania, Australia | Primary and high school students (aged 7-18) | To explore children’s climate attitudes and understandings through a detailed thematic analysis of questions about climate change submitted to Curious Climate Schools. | Nil | Written dialogue (submitted questions)  N = 1500 | Anxiety, holistic wellbeing, climate worry, frustration, existential anxiety, emotional landscape, climate action | Science communication and education |
| 77 | Rodriguez Quiroga (2024)^108^ | Argentina and Spain | University students aged 15-57 (mean age 22.6) and participants aged 14-89 (mean age 40.80) | To increase the availability of the Hogg Eco-Anxiety Scale (HEAS) by translating the measure into Spanish and validating in non-English speaking populations. | Nil | Survey  Spanish participants (N = 548) and Argentinian participants (N = 990) | Eco anxiety, climate anxiety, climate concerns, emotional responses, climate worry, tense, terrified, mental wellbeing, pro-environmental behaviour | Nil |
| 78 | Murphy (2024)^109^ | Non-specific | Non-specific | To describe how children’s authors are increasingly drawing on tropes of ecological fiction in order to respond to the current climate crisis and offer young readers models of activism that will address this major global issue. | Nil | Essay | Ecological concerns, healing and grieving, ecoglobalist affect, ecological grief, frustration, eco anxiety, climate anxiety, biophilia, activism, personal responsibility | Social justice |
| 79 | Romano et al. (2024)^110^ | Northern and Southern Italy | High school students | To investigate the relationship between climate anxiety and the likelihood of participating in pro-environmental  movements via getting information through social media on pro-environmental content. | Social identity model for collective action | Survey  N = 480 | Climate anxiety, eco-paralysis, anger, environmental engagement, risk perceptions | Avoidance and escape-oriented coping strategies, activism |
| 80 | Pihkala (2024)^111^ | Nonspecific | Non-specific, includes young people | To provide important information regarding encounters between children and adults in relation to climate change and other environmental crises. Also, to explore various kinds of loss and grief that people, especially children and youth, may experience in relation to climate change. | Maturational loss, psychodynamic approach, post-traumatic growth | Review | Climate anxiety, climate grief, climate distress, ecological grief, emotional responses, eco anxiety, solastalgia, loss, chronic sorrow, moral injury, post-traumatic growth, global-scale worry, climate action, concerns about the future, beliefs about government, climate change maturity | Spaces for emotional expression, culture of care, multiple strategies, social support, recognising and validating loss |
| 81 | Olson et al. (2024)^112^ | Australia | Non-specific, includes young people | To present a multi-method qualitative text and discourse analysis of Australian online news articles published in 2022 reporting on emotions and our ecological future. | Emotional discourses,  Theories of collective emotions | Mixed-methods qualitative and discourse analysis | Eco anxiety, emotional sequelae from climate change (depleting and guilt-invoking), climate anxiety, anger, helplessness, pre- and post-traumatic stress disorders associated with eco-anxiety, affective entanglements with climate change, anxiety, emotional responses, social and moral emotions, sociopolitical action, worldviews and meaning systems, including spiritual and/or existential aspects | Multiple strategies,  Emotional regulation, Actism |
| 82 | Ágoston et al. (2024)^113^ | Hungary | Adults from the general population. Sample two included high school students and one of their parents | To examine the frequency of different pro-environmental behaviours across age groups segmented by decades, and thereby detect more subtle age differences. Also, to examine behaviors by parents and children living in the same household, which could shed light on generational differences and socialization effects. Lastly, to explore latent profiles of eco-emotions (anxiety, guilt, grief) and behavior, and to compare latent profiles across age groups. |  | Survey  Sample 1 (N = 4,685), Sample 2 (N = 112) | Climate/eco-emotions, climate anxiety, ecological grief, eco-guilt, climate worry, eco anxiety, depression, climate concerns, maladaptive responses, pro-environmental behaviour, awareness, self-efficacy, climate change beliefs | Nil |
| 83 | Ndetei et al. (2024)^114^ | Kenya, Africa | High school students (mean age 16.13) | To explore the perceived impact of climate change on mental health and suicidality in Kenyan high school students. |  | Survey  N = 2,596 | Mental health, PTSD, suicidality, depression, emotional responses, anxiety, climate/eco-emotions | Nil |
| 84 | Trost et al. (2024)^115^ | Germany | Psychotherapists. Patients were mainly young adults | To quantify psychotherapists’ experiences with patients reporting climate change-related concerns and their views on dealing with this topic in psychotherapy. |  | Survey  (N = 573) | Mental health, traumatic stress, PTSD, anxiety, depression, climate/eco-emotions, emotional responses, climate concerns, climate change-related reactions, pro-environmental behaviour | Therapy |
| 85 | Fekih-Romdhane et al. (2024)^116^ | Lebanon | Young adults aged 18-35 | To examine the relationship between climate change anxiety and psychotic experiences, and to test the theoretically-driven hypothesis that death anxiety acts as a mediator in this relationship. | Vulnerability stress model of psychosis | Survey  (N = 596) | Climate anxiety, mental health, death anxiety, climate concerns, functional impacts on daily life | Nil |
| 86 | Veijonaho et al. (2024)^117^ | Helsinki, Finland | Adolescents aged 11–15 | To investigate adolescents’ climate change distress and climate denialism profiles with two cohorts, and to study the explanatory similarity of the subgroups regarding general well-being and pro-environmental behavior. |  | Survey  (N = 2020) | Climate distress, subjective wellbeing, climate concerns, climate worry, climate anxiety, mental health, eco-anxiety, pro-environmental behaviour, climate change beliefs, awareness, identity | Constructive vs. unconstructive coping, values |
| 87 | Hieronimi et al. (2024)^118^ | Germany | Professionals providing care for children and adolescents | To assess if the relevance of extreme weather events associated with mental health impairments in children and adolescents by caregiving professionals differs depending on if their environment is more affected, and if their environment is urban vs. rural. |  | Survey  (N = 648) | Mental health, traumatic stress, PTSD, suicidality, aggression, emotional responses, pro-environmental behaviour, risk perceptions, awareness | Nil |
| 88 | Luk & Longman (2024)^119^ | Northern Rivers, New South Wales, Australia | Young people aged 16-25 | To explore young people's experiences of catastrophic flooding in the Northern Rivers area of northern NSW in 2017 and its effect on their mental health. |  | Survey  Baseline (N = 125), Open-text responses (N = 81) | Mental health, depression, anxiety, PTSD, psychological distress, eco-anxiety, pre-traumatic stress, emotional responses, emotions and impacts on everyday life, resilience, trust vs. distrust, financial strain | Community participation |
| 89 | Spitzer et al. (2024)^120^ | United States | High school students | To evaluate the potential for self-determination theory to guide efforts to communicate with youth about climate change. Specifically, to examine how needs-(mis)aligned communication styles affect adolescents’ emotional responses to information about climate change. | Self-determination theory | Experiment  Study 1 (N = 141), Study 2 (N = 270) | Mental wellbeing, anxiety, anxiety, emotional responses, climate anxiety, mental ill-health, hypervigilance, hope | Climate conversations |
| 90 | Vercammen, Oswald & Lawrence (2023)^121^ | United Kingdom | Young people aged 16-24 | To explore, in young UK residents who experience ‘climate distress’, who is more likely to be affected and how distress presents itself. |  | Mixed-methods qualitative and quantitative  N = 539 | Climate worry, climate distress, mental health, climate anxiety, psychological adaptation to climate change, functional impacts on daily life, practical anxiety, climate hope, eco-guilt, climate/eco-emotions, pro-environmental behaviour, agency | Multiple strategies  Constructive hope |
| 91 | Pinchoff et al. (2023)^122^ | Mexico | Young adults aged 15-24 | To understand how recent climate hazards (in the last 12 months) are associated with common mental health disorders (CMD), a combination of reported depression and anxiety symptoms. Also, to explore how such outcomes are associated with feelings of individual agency and concern related to climate change. |  | Survey  Males (N = 46, 074), females (N = 65,742), non-binary/trans (N = 2,154) | Mental health, general psychological distress, PTSD, anxiety, depression, suicidality, climate dread, climate anxiety, frustration, betrayal, autonomy, agency | Nil |
| 92 | Coffey et al. (2021)^10^ | Non-specific | Non-specific, includes young people | To understand how eco-anxiety was operationalized in the existing literature, and to identify the key characteristics of eco-anxiety. |  | Review | Eco anxiety, environmentally-induced distress, ecological grief, solastalgia, eco-angst, environmental distress, climate change anxiety, ecological stress, dissatisfaction with government, anxiety, positive and negative emotions, eco-depression, eco-anger, climate/eco-emotions | Nil |
| 93 | Tito et al. (2024)^123^ | Philippines | Non-specific, includes young people | To provide an overview of current research knowledge and research gaps regarding the impacts of climate change outcomes on Filipinos’ mental health and well-being. |  | Review Articles = 32 | Mental health, PTSD, depression, anxiety, sleep disorders, acute stress disorder, eco-anxiety, aggression, climate grief, resilience, post-traumatic growth | Multiple strategies, Psychological first aid, Cooperation, Access to healthcare, Culturally adapted cognitive and behavioral therapy, Helping each other out, Reserving energy for essential purpose, Religious values |
