# Supplementary material for: Conceptualizing Young People's Experiences of Climate Change Awareness: A Narrative Review
Source: Ann N Y Acad Sci. 2025 Oct 26;1554(1):45–65. doi: 10.1111/nyas.70114 (PMC12728334; doi:10.1111/nyas.70114)
Supplement: Supplementary file 3 — Table S3. Most frequently referenced coping strategies to manage young people's experiences in response to awareness of the climate crisis. [file NYAS-1554-45-s001.docx]

Supporting Table S3. Most frequently referenced coping strategies to manage young people’s experiences in response to awareness of the climate crisis

| **Top 8 Referenced Strategies, (Number of Papers)** | **Example description provided in paper (reference)** |
| --- | --- |
| **Meaning focused- coping (13)** | “Meaning-focused coping involves reflecting upon the meaning and benefits of a difficult situation. Because it tends to engender positive emotions like trust and hope, it can be considered a psychological resource” (Voltmer & von Salisch, 2024, p. 6)^49^ |
|  |  |
| **Problem-Focused Coping (10)** | “Problem-focused coping entails attempts to solve the problem. For example, one may think about different things one can do to deal with climate change” (Wullenkord & Ojala, 2023, p.3)^101^ |
| **Community Participation (9)** | “Enhancing community connectedness through promotion of community events and activities, especially in the aftermath of floods, can serve as a protective factor” (Luk & Longman, 2024, p. 350)^119^ |
| **Young people’s Empowerment and Policy Advocacy (9)** | “...empowering and encouraging action and campaigns from youths” (Kaligis, 2023, p. 251)^83^ |
| **Science Communication and Education (9)** | “Action-oriented climate change education may help educators develop a collective sense of purpose in their work, while also developing a sense of empathy with their students' anxieties and worries about the future” (Mateer, 2023, p. 6)^82^ |
| **Nature-Based Solutions (6)** | “Engaging with the natural environment” (Koder et al., 2023, p. 10)^52^ |
| **Avoidance and escape-oriented coping strategies (5)** | “Defensive, self-protective strategies to suppress, deny, or avoid (the source of) uncomfortable feelings” (Romano et al., 2024)^110^ |
| **Spaces for Emotional Expression (5)** | “...a safe space for individuals to express how they truly feel without fear of judgment or retribution” (Ettinger et al., 2023, p.10)^105^ |
